# Physical activity enhances the improvement of body mass index and metabolism by inulin: a multicenter randomized placebo-controlled trial performed in obese individuals

**DOI:** 10.1186/s12916-022-02299-z

**Published:** 2022-03-30

**Authors:** Julie Rodriguez, Audrey M. Neyrinck, Maxime Van Kerckhoven, Marco A. Gianfrancesco, Edith Renguet, Luc Bertrand, Patrice D. Cani, Nicolas Lanthier, Miriam Cnop, Nicolas Paquot, Jean-Paul Thissen, Laure B. Bindels, Nathalie M. Delzenne

**Affiliations:** 1 Metabolism and Nutrition Research Group, Louvain Drug Research Institute, UCLouvain, Université catholique de Louvain, Brussels, Belgium; 2 Laboratory of Diabetology, Nutrition and Metabolic Disease, Université de Liège, Liège, Belgium; 3 Pole of Cardiovascular Research, Institut de Recherche Expérimentale et Clinique, Université catholique de Louvain, Brussels, Belgium; 4 WELBIO- Walloon Excellence in Life Sciences and BIOtechnology, UCLouvain, Université catholique de Louvain, Brussels, Belgium; 5 Laboratory of Hepatogastroenterology, Institut de Recherche Expérimentale et Clinique, UCLouvain, Université catholique de Louvain, Brussels, Belgium; 6 ULB Center for Diabetes Research, Université Libre de Bruxelles, and Division of Endocrinology, Erasmus Hospital, Brussels, Belgium; 7 Pole of Endocrinology, Diabetes and Nutrition, Institut de Recherche Expérimentale et Clinique, UCLouvain, Université catholique de Louvain, Brussels, Belgium

**Keywords:** Obesity, Gut microbiota, Prebiotic, Physical activity, Metabolism

## Abstract

**Background:**

Dietary interventions targeting the gut microbiota have been proposed as innovative strategies to improve obesity-associated metabolic disorders. Increasing physical activity (PA) is considered as a key behavioral change for improving health. We have tested the hypothesis that changing the PA status during a nutritional intervention based on prebiotic supplementation can alter or even change the metabolic response to the prebiotic. We confirm in obese subjects and in high-fat diet fed mice that performing PA in parallel to a prebiotic supplementation is necessary to observe metabolic improvements upon inulin.

**Methods:**

A randomized, single-blinded, multicentric, placebo-controlled trial was conducted in obese participants who received 16 g/day native inulin versus maltodextrin, coupled to dietary advice to consume inulin-rich versus -poor vegetables for 3 months, respectively, in addition to dietary caloric restriction. Primary outcomes concern the changes on the gut microbiota composition, and secondary outcomes are related to the measures of anthropometric and metabolic parameters, as well as the evaluation of PA. Among the 106 patients who completed the study, 61 patients filled a questionnaire for PA before and after intervention (placebo: *n* = 31, prebiotic: *n* = 30). Except the dietitian (who provided dietary advices and recipes book), all participants and research staff were blinded to the treatments and no advices related to PA were given to participants in order to change their habits. In parallel, a preclinical study was designed combining both inulin supplementation and voluntary exercise in a model of diet-induced obesity in mice.

**Results:**

Obese subjects who increased PA during a 3 months intervention with inulin-enriched diet exhibited several clinical improvements such as reduced BMI (− 1.6 kg/m^2^), decreased liver enzymes and plasma cholesterol, and improved glucose tolerance. Interestingly, the regulations of *Bifidobacterium, Dialister*, and *Catenibacterium* genera by inulin were only significant when participants exercised more. In obese mice, we highlighted a greater gut fermentation of inulin and improved glucose homeostasis when PA is combined with prebiotics.

**Conclusion:**

We conclude that PA level is an important determinant of the success of a dietary intervention targeting the gut microbiota.

**Trial registration:**

ClinicalTrials.gov, NCT03852069 (February 22, 2019 retrospectively registered).

**Supplementary Information:**

The online version contains supplementary material available at 10.1186/s12916-022-02299-z.

## Background

The interest of a prebiotic approach in the modulation of gut microbiota for the management of obesity and/or associated metabolic disorders is emerging from recent studies [1, 2]. Prebiotics are defined as “substrates that are selectively utilized by host microorganisms conferring a health benefit” [3]. Prebiotics such as Inulin-type fructans (ITF) have largely been studied in the context of obesity and related metabolic disorders. Among the health benefits, various studies highlighted in rodent models of obesity that ITF intake improves gut barrier function [4], low-grade inflammation [4, 5], fat mass accumulation [6–8], glucose intolerance [5, 6, 9], postprandial hypertriglyceridemia [10], hepatic steatosis [6, 7, 11, 12], and endothelial dysfunction [13]. Intervention studies in humans also confirm the interest of ITF supplementation in the management of metabolic disorders and obesity [14, 15]. Intriguingly, the metabolic improvement following nutritional interventions such as dieting is quite variable from one patient to the other [16]. Recent studies pointed out individual factors, such as the initial gut microbiota composition, usual dietary fiber intake, or medications (such as metformin), that could influence the metabolic response to ITF in obese individuals [12, 15]. In this paper, we addressed the hypothesis that physical activity (PA) is another condition that potentially affects both the gut microbiota and prebiotic efficacy. Indeed, exercise training is a well-known cost-effective intervention that can improve or prevent the development of metabolic disorders [17, 18]. In addition, the gut microbiota composition differs in professional athletes compared to the gut microbiota from sedentary people [19, 20]. In vivo experiments have shown that exercise alters the gut microbiota composition in normal, obese or diabetic mice, with some differences according to the metabolic status of mice [21–24]. An elegant study identified in prediabetic individuals that the gut microbiota was an important factor influencing the improvement of glucose metabolism and insulin sensitivity during exercise [25]. Indeed, responders and non-responders to exercise exhibited differences in their gut microbiota composition. Moreover, the metabolic benefit to exercise observed in the responder group was transferable into mice using fecal microbial transplantation. In addition, a previous report demonstrated that 4-weeks of inulin-propionate ester, in combination with a moderate intensity exercise training program, increased the amount of fat oxidized in overweight women. This study suggested that combining PA with inulin-propionate ester is a good strategy for improving lipids metabolism in overweight subjects [26].

While exercise training and ITF have been shown to impact the gut microbiota independently and are each associated with beneficial health effects, we investigated in both preclinical and clinical interventions, the interaction between inulin intake as prebiotic intervention and PA in the context of obesity. Using data obtained in a cohort of obese individuals treated with inulin supplements coupled to inulin-rich vegetables versus placebo, we addressed two questions. First, does inulin intervention modify PA levels in obese patients? Second, do changes in PA level during the intervention impact on the effect of inulin on gut microbiota and metabolism? In parallel, we designed an intervention study with voluntary exercise and/or inulin supplementation in HF-fed mice to investigate the molecular mechanisms behind the modulation of metabolic and microbial outcomes linked to combined PA and inulin intervention.

## Methods

Supplementary protocols and complete procedures are described in the online additional file.

### Subjects

This study was approved by the “Comité d’éthique Hospitalo-facultaire de Saint-Luc”. Written informed consent was obtained from all participants before inclusion. The trial protocol was published on protocols.io (dx.doi.org/10.17504/protocols.io.baidica6), and the trial was registered at ClinicalTrials.gov under identification number NCT03852069.

#### Screening and inclusion

Male and female subjects were recruited in three university hospitals in Belgium (Hôpital Erasme in Brussels, Centre Hospitalier Universitaire in Liege and Cliniques universitaires Saint-Luc in Brussels). This study was a 3-month long, multicentric, single-blind, and placebo-controlled trial. The inclusion criteria were as follow: body mass index (BMI) > 30 kg/m^2^, aged 18 to 65 years, Caucasian ethnicity, and the presence of at least one obesity-related metabolic disorder (including prediabetes/diabetes, dyslipidemia, hypertension, or elevated hepatic enzymes suggestive of metabolic associated fatty liver disease “MAFLD”). The exclusion criteria included the use of antibiotics, pro/prebiotics, dietary fiber supplements, or any molecules that modifies intestinal transit time within 6 weeks enrolment, pregnancy in progress or planned within 6 months, presence of psychiatric problems and/or use of antipsychotics, following a special diet (e.g., vegetarian, vegan), recent (< 6 weeks) or ongoing diets (e.g., high-protein, high-fiber diet), excessive alcohol consumption (more than 3 glasses/day), type 1 diabetes, and general dislike for vegetables.

#### Randomization

Following the screening step, subjects were randomly assigned to the prebiotic or placebo arm. (random sequence generated using MS Excel®, simple randomization). Randomization sequences were not revealed to the study staff.

#### Blinding

All participants and research staff (except dietitians who provided dietary advices and recipes books) were blinded to the treatments. One person was in charge of enrolling and assigning participants to interventions, in each center.

#### Outcomes

The primary outcome of this trial was the effect of the prebiotic intervention on the gut microbiota composition. Secondary outcomes of this trial concern the effect of prebiotic intervention on anthropometric and clinical parameters but also on the evaluation of physical activity.

CONSORT checklist is provided in supplemental information.

#### Dietary intervention, energy, and nutrient intake

Subjects were asked to consume either16 g/day of native inulin for the prebiotic group (extracted from chicory root, Cosucra, Belgium) or 16 g/day of maltodextrin for the placebo group (Cargill, Belgium), provided in identical packaging, for a period of 3 months. Empty and unused packets were returned to measure compliance. In addition to inulin or maltodextrin intake, participants received a cookbook with recipes based on vegetables either rich (for prebiotic group) or poor (for placebo group) in fructans. Participants were thus advised to consume at least one meal a day with a recipe from this cookbook. Participants received before, and monthly during the protocol, dietary advices from a dietitian in order to reduce their energy intake during the intervention. Energy and nutrient intake were calculated from the one-week recall using the Nubel Pro program (Nubel asbl, Brussels, Belgium). A telephone follow-up was performed three times during the intervention (after 2 weeks, 1 and 2 months).

#### IPAQ questionnaire

At baseline and at the end of the protocol, 61 participants had a meeting with a unique person by center, in order to fill a long form of International PA Questionnaire (IPAQ). The IPAQ long form asks details about the specific types of activities undertaken within the four main domains (leisure time PA, domestic and gardening (yard) activities, work-related PA and transport-related PA). Computation of the total scores for the long form requires summation of the duration (in minutes) and frequency (days) for all the types of activities in all domains. Results allowed us to classify different levels of PA proposed by the questionnaire (total, low-moderate or high-intensity PAs). Among the 106 patients who completed the study, 61 patients filled the questionnaire for PA before and after intervention (placebo: *n* = 31, prebiotic: *n* = 30). After evaluation of PA according to this questionnaire, there were 19 participants who voluntarily increased their PA during the protocol in the maltodextrin group and 14 in the inulin group. 12 participants in the maltodextrin arm and 16 participants in the inulin arm did not change or voluntarily decreased their PA during the intervention.

### Mice procedure

Forty-five mice (9-week old C57BL/6 J male) were purchased from Janvier Labs (Le Genest St Isle, France). Mice were individually housed in a controlled environment (12-h daylight cycle) with free access to food and water. The experiment was approved by and performed following the guidelines of the local ethics committee for animal care of the Health Sector of Université catholique de Louvain under the specific agreement number 2017/UCL/MD/005. Housing conditions were as specified by the Belgian Law of 29 May 2013 regarding the protection of laboratory animals (Agreement no LA 1230314). Every effort was made to minimize animal pain, suffering, and distress.

The acclimatization period lasted one week with a standard diet (Research Diet Inc., New Brunswick, NJ, USA). Two independent experiments were performed. Randomization of mice into five groups was done based on body weight to minimize baseline differences. Experimental groups consisted in one group of mice fed with a low-fat diet (LF: *n* = 10; 10% kcal from fat, D12450, Ssniff, Soest, Germany) and four groups of mice fed with a high-fat diet (HF, 45% kcal from fat, D12451, Ssniff, Soest, Germany). Mice fed with a HF were divided in four additional groups: (1) a group fed a HF only (HF: *n* = 8); (2) a group fed a HF and receiving 0.3 g/day per mouse of native inulin (Fibruline®, Cosucra, Pecq, Belgium) in the drinking water (INU: *n* = 9) ; (3) a group fed a HF and receiving a running wheel coupled to an electronic device for measuring the voluntary exercise performed (Ex: *n* = 9); and (4) a group fed a HF receiving both inulin supplementation and the running wheel (INU + Ex: *n* = 9). There was no exclusion of mice during the procedure. Running wheel was coupled with an odometer for recording continuously the activity of mice (kilometers and time spent in the wheel). Odometers were tested for reliability of time and distance covered. The cages were visually checked three times a week to ensure that odometers correctly recorded mice activity. To ensure a same enrichment of environment for all mice, the other groups of mice also received a locked wheel in their cage.

At the sixth week, a 24-h feces collection was performed.

At the seventh week, an oral glucose tolerance test (OGTT) was performed on 6 h fasted-mice. Glucose was administered orally (2 mg/g of body weight, 50% glucose solution), and blood glucose levels were determined using a glucose meter (Roche Diagnostics) on blood collected from the tip of the tail vein both before (30 min and 0 min) and after glucose administration (15, 30, 60, 90, 120 min). Twenty microliters of blood were sampled 30 min before and 15 min after the glucose load to assess plasma insulin concentrations.

At the eighth week, running wheels in the two exercised groups were also locked 24 h before the end of the protocol. After 6 h of fasting, mice were anesthetized using isoflurane gas (Abbot, Ottignies, Belgium). Blood from cava vein was harvested in EDTA tubes. Plasma was immediately collected after centrifugation (12 000 × *g* for 3 min) and stored at − 80 °C for biochemical analysis. Mice were necropsied after cervical dislocation. Liver, brown and white adipose tissues (epididymal, visceral, and subcutaneous), *gastrocnemius* muscles, cecal content, and intestinal tissues were dissected and immersed in liquid nitrogen before storage at − 80 °C.

ARRIVE checklist is provided in supplemental information.

### Gut microbiota analysis

The raw sequencing data can be accessed in the Sequence Read Archive with the SRA accession number: PRJNA595949 (cohort subset) and PRJNA721281 (mouse subset) [27].

#### Cohort subset

Stool samples were available for 59 patients (*n = 11 for maltodextrin, n = 19 for maltodextrin + PA; n = 16 for inulin and n = 13 for inulin + PA*). Stool samples were collected at baseline and at the end of the 3-month of intervention and stored at room temperature with a DNA stabilizer (Stratec biomolecular, Berlin, Germany) for maximum 3 days, and then transferred to − 80 °C for the analysis of the gut microbiota composition. Genomic DNA was extracted from feces using a PSP® spin stool DNA kit (Stratec biomolecular, Berlin, Germany).

#### Mouse subset

DNA was extracted from the mouse cecal content using a QIAamp DNA Stool Mini Kit (Qiagen, Hildren, Germany) including a bead-beating step.

For mouse and human subsets, subsequent bioinformatics and biostatistics analyses were performed *in house* as previously described [12].

Initial quality filtering of the reads was performed with the Illumina Software, yielding an average of 96943 pass-filter clusters per sample for the human cohort and 130174 for the mouse cohort. A subset of 25000 reads for the human cohort and 34000 reads for the mouse cohort were randomly selected using Mothur v1.25.0 [28] to avoid large disparities in the number of sequences. Taxonomic prediction was performed using the nbc_tax function [29], an implementation of the RDP Naive Bayesian Classifier algorithm [30]. Taxonomy for significant ASV (amplicon sequence variants) was also confirmed using the EzBioCloud 16S database. Alpha-diversity and beta-diversity indexes were calculated using QIIME [31]. PcoA plot of the beta-diversity indexes were visualized using the R software. Barplots for phylum was visualized on the GraphPad Prism version 8.0 software.

### Statistical analysis

#### Gut microbiota analysis

For gut microbiota analysis, data are expressed as the mean percentage of relative abundance ± SEM. A Kruskal–Wallis ANOVA test was performed for detecting significant differences for taxa and ASV between groups, followed by Dunn’s multiple comparisons test, using R software (version 3.5.1) and the FSA package. *p*-value of the Kruskal-Wallis test was adjusted (*q*-value, significant if *q* < 0.05) to control for the false discovery rate (FDR) for multiple tests according to the Benjamini and Hochberg procedure [32]. To avoid analyzing spurious sequences, only ASV with at least one sample showing a relative abundance of more than 0.1% were kept.

In the cohort and mouse experiments, α-diversity indexes were analyzed with a two-way ANOVA, followed by Tukey’s multiple comparison test (significant if *p* < 0.05). For β-diversity index, a Monte-Carlo rank test was performed.

Association between the variation of genera or ASV significantly regulated in the cohort and significant changes in metabolic outcomes was assessed by Spearman’s correlation tests. A significance level of *p* < 0.05 was adopted for all analyses.

#### In the cohort

Clinical outcomes were analyzed using mixed model effects (with PA and inulin supplementation as fixed effects and patients and hospitals as random effects) using the JMP Pro 14 software.

#### Mice experiment

Variables were analyzed with a two-way ANOVA, performed in the four groups of mice fed a high-fat diet, followed by Tukey’s multiple comparisons test. Within-groups variances were compared using Bartlett’s test. If variances were significantly different between groups, values were normalized by Log transformation before proceeding to the analysis.

## Results

### PA maximizes the improvement of clinical outcomes upon inulin intervention in obese individuals

**Fig. 1:**
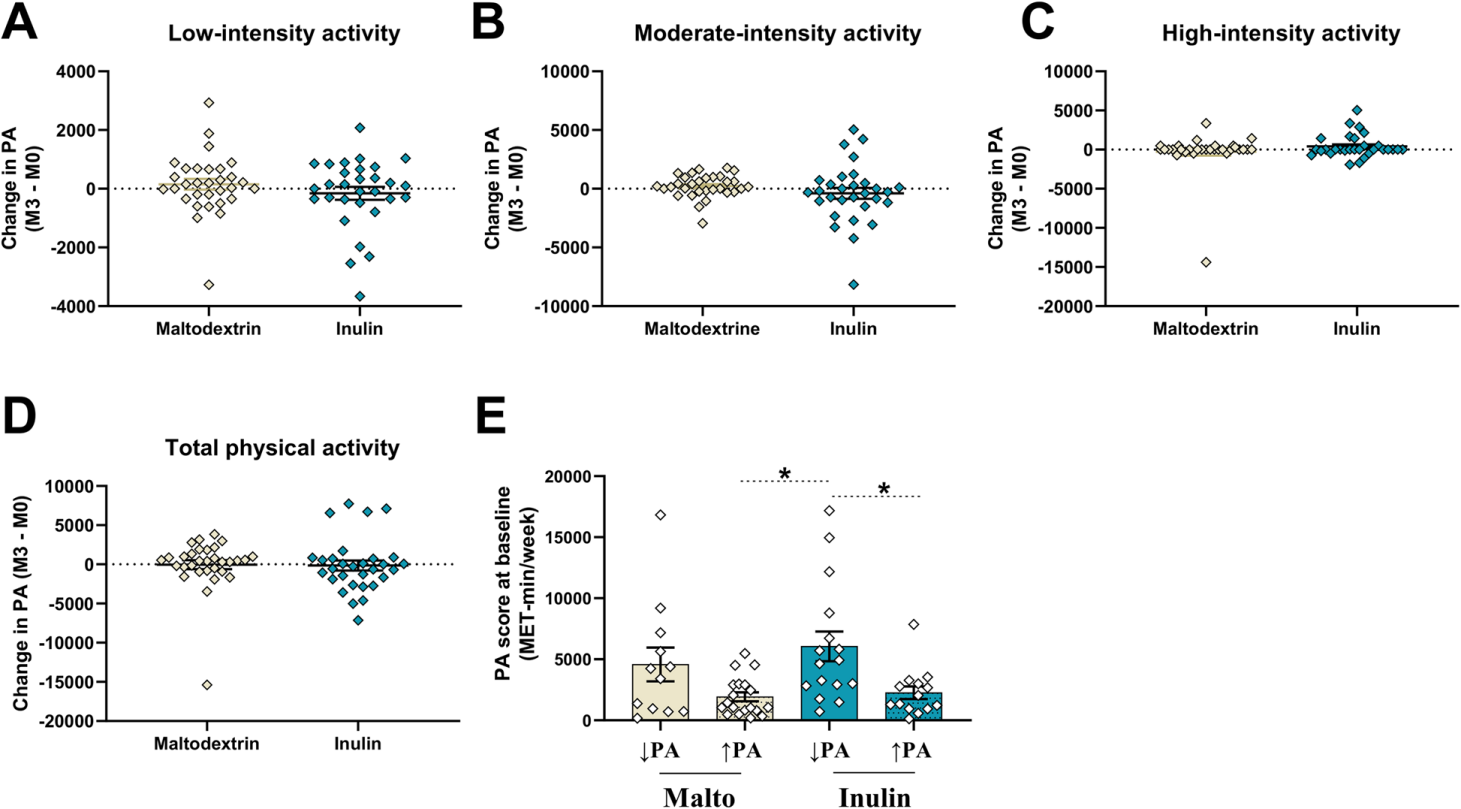
PA performed by participants during the intervention protocol. **A**–**D** Differences (month 3—baseline) in low-intensity, moderate-intensity, high-intensity, and total PA performed by participants throughout the protocol (*n* = 31 for placebo maltodextrin group, *n* = 30 for inulin group). **E** IPAQ score for PA performed by participants at baseline (before intervention). A one-way ANOVA followed by Dunn’s test for multiple comparisons was applied for comparisons between groups. (*n* = 12 for maltodextrin, *n* = 19 for maltodextrin with increased PA, *n* = 16 for inulin and *n* = 14 for inulin with increased PA)

We used the data collected in a cohort of obese patients enrolled in a large multicenter clinical intervention aiming to study the impact of 3-month inulin supplementation (16 g/day) or maltodextrin (16 g/day) as placebo, combined with dietary advice to consume vegetables enriched in ITF (for inulin arm) versus vegetables poor in inulin (for placebo arm) [15]. Within this cohort, a subset of patients (*n* = 61) recruited in two different hospitals filled an IPAQ questionnaire, at both baseline and the end of the protocol, in order to evaluate the PA level for each participant during the protocol (Figure Flow diagram). The participants did not receive advice to change PA during the study. At the end of the study, there were no significant changes in low-, moderate-, and high-intensity and total PA in the participants from both groups (*n* = 31 for maltodextrin and *n* = 30 for inulin, Fig. 1A–D). However, within each group, some participants voluntarily increased their activity whereas other decreased or did not change it. In the placebo group, 61% of subjects had increased total PA at the end of the study compared to 47% in the inulin group (Additional file: Table 1). This allowed us to separate the cohort in four groups (maltodextrin or inulin groups with decreased/unchanged PA and maltodextrin or inulin groups with increased PA, Additional File: Table 1), in order to evaluate possible additional effects of voluntarily increased PA and inulin supplementation on gut microbiota composition and clinical outcomes. Participants exhibited significantly different PA habits prior to intervention (Fig. 1E), which were associated with differences in BMI and total body fat (Additional file: Table 2).

Subjects from each group reduced their energy intake similarly in response to the dietary advice (Table 1). At the end of the intervention, inulin reduced body weight and BMI, and to a greater extent in participants who increased PA (Table 1). This latter group exhibited a reduction in waist circumference and waist/hip ratio. PA globally improved visceral fat and tended to improve systolic blood pressure. In addition, inulin improved liver stiffness and plasma AST levels, independently of PA changes (Table 1). Interestingly, plasma gGT and total cholesterol were significantly reduced only in participants receiving inulin who increased PA. This group also had significantly lower fasting insulin and improved HOMA-IR (homeostasis model assessment of insulin resistance insulin sensitivity index), HOMA-ISI (HOMA insulin sensitivity index), and Matsuda indexes (measuring insulin sensitivity). Finally, this group also had decreased DPP-IV (dipeptidyl-peptidase IV) activity. To conclude, PA had little impact in the placebo group whereas it significantly improved metabolic parameters in inulin-treated participants.

**Table 1:** Changes in anthropometric and clinical outcomes in obese patients receiving prebiotic or placebo for 3 months, according to their PA during the protocol

	Malto ↓PA		Malto ↑PA		Inulin ↓PA		Inulin ↑PA		***Between groups comparisons (mixed model)***		
	Baseline	3 months	Baseline	3 months	Baseline	3 months	Baseline	3 months	*Inulin*	*PA*	*Interaction*
***Energy and nutrients intake***											
Energy (kcal)	**2177.5 ± 158.4**	1838.3 ± 116.4*†	**1873.3 ± 73.9**	1612.6 ± 67.7*†	**1996.8 ± 90.1**	1693.8 ± 90.0 *	**1953.5 ± 123.7**	1544.9 ± 86.4*†	ns	ns	ns
Protein (g)	**99.8 ± 6.5**	87.4 ± 4.6*	81.9 ± 3.0	79.2 ± 3.0	86.9 ± 3.5	78.0 ± 3.4	**80.7 ± 4.5**	71.9 ± 3.3*	ns	ns	ns
Lipid (g)	**81.8 ± 8.2**	63.2 ± 6.3*	**76.1 ± 4.8**	60.3 ± 3.6*†	**80.1 ± 7.0**	63.3 ± 4.2*	**73.8 ± 6.0**	55.6 ± 2.9*	ns	ns	ns
Carbohydrates (g)	235.2 ± 17.2	207.5 ± 13.1	**196.5 ± 10.7**	170.5 ± 12.4*	**217.5 ± 10.0**	186.9 ± 13.5*	**220.5 ± 20.0**	172.5 ± 15.7*†	ns	ns	ns
Fiber (g)	22.8 ± 1.6	22.7 ± 1.8	20.3 ± 1.3	21.7 ± 1.5	23.4 ± 2.5	25.5 ± 2.2	21.4 ± 3.0	25.1 ± 2.2	ns	ns	ns
***Anthropometric outcomes***											
BMI	35.9 ± 1.8	35.7 ± 1.8	33.3 ± 0.5	32.9 ± 0.5	35.1 ± 0.9	34.6 ± 1.0	**39.7 ± 1.7**	38.1 ± 1.8*†	**0.012**	ns	**0.044**
Weight (kg)	105.6 ± 3.5	104.9 ± 3.5	98.0 ± 3.2	96.9 ± 3.2	96.4 ± 3.2	95.0 ± 3.5	**114.7 ± 6.8**	110.2 ± 7.1*†	**0.016**	ns	**0.04**
Total body Fat (kg)	40.1 ± 3.7	39.9 ± 3.4	35.0 ± 2	35.0 ± 1.8	37.5 ± 2.4	36.2 ± 2.5	46.0 ± 2.7	44.7 ± 3.2	ns	ns	ns
Waist (cm)	116.7 ± 3.2	115.0 ± 2.7	**109.0 ± 2.0**	106.2 ± 1.8*	109.7 ± 2.3	108.5 ± 3.0	**119.9 ± 5.1**	116.2 ± 5.5*†	ns	ns	ns
Waist/Hip ratio	0.98 ± 0.03	0.97 ± 0.03	0.96 ± 0.02	0.95 ± 0.02	0.94 ± 0.02	0.93 ± 0.02	**0.94 ± 0.02**	0.92 ± 0.02*	ns	ns	ns
Visceral fat (cm^2^)	292.2 ± 40.3	295.2 ± 45.2	225.7 ± 22.5	218.2 ± 20.6	205.4 ± 19.9	207.2 ± 21.8	231.4 ± 29.6	217.2 ± 28.7	ns	**< 0.001**	ns
Systolic blood pressure (mm Hg)	140.5 ± 5.3	134.2 ± 6.4	**138.6 ± 4.3**	130.5 ± 3.1*	134.3 ± 2.8	135.8 ± 3.1	135.8 ± 3.8	127.2 ± 4.6	ns	0.087	ns
Diastolic blood pressure (mm Hg)	87 ± 3.8	85.9 ± 3.4	84.4 ± 2.3	81.2 ± 1.7	86.1 ± 2.4	82.6 ± 3.5	87.8 ± 2.6	83.9 ± 3.7	ns	ns	ns
Liver stiffness (kPa)	5.9 ± 1.0	6.5 ± 0.9	6.8 ± 0.9	6.9 ± 1.0	5.6 ± 0.6	4.7 ± 0.2	6.5 ± 1.4	5.5 ± 1.0	**0.036**	ns	ns
***Clinical outcomes***											
AST (U/l)	30.3 ± 3.6	33.3 ± 4.4	28.4 ± 3.4	28.2 ± 3.2	25.7 ± 3.1	23.8 ± 2.4	**21.7 ± 1.2**	19.6 ± 1.6*	**0.023**	ns	ns
ALT (U/l)	40.5 ± 5.9	46.7 ± 9.3	45.0 ± 7.7	45.2 ± 8.1	32.9 ± 5.2	33.3 ± 4.5	29.4 ± 2.8	27.0 ± 3.3	ns	ns	ns
gGT (U/l)	61.8 ± 14.8	52.0 ± 10.9	40.8 ± 7.0	40.0 ± 5.9	32.8 ± 5.2	29.9 ± 4.3	**39.8 ± 7.6**	31.2 ± 5.8*†	ns	ns	ns
Total cholesterol (mg/dl)	172.3 ± 9.2	165.5 ± 8.0	199.3 ± 13.6	194.6 ± 13.5	209.7 ± 11.7	217.0 ± 13.9	**192.9 ± 14.2**	176.1 ± 11.1*	**0.031**	ns	**< 0.001**
LDL cholesterol (mg/dl)	95.1 ± 11.7	86.2 ± 9.2	126.9 ± 11.5	122.0 ± 11.2	131.1 ± 10.0	135.8 ± 12.3	114.8 ± 13.6	106.6 ± 11.1	ns	ns	**< 0.001**
HDL cholesterol (mg/dl)	45.4 ± 2.8	48.1 ± 3.8	49.1 ± 2.4	47.2 ± 2.1	45.9 ± 2.6	47.0 ± 2.9	49.2 ± 3.7	47.1 ± 3.5	ns	**0.0165**	ns
Triglycerides (mg/dl)	172.6 ± 22.2	166.5 ± 29.9	143.8 ± 19.1	150.3 ± 21.2	172.2 ± 19.2	183.9 ± 23.4	192.9 ± 61.8	126.4 ± 19.2	ns	ns	ns
HbA1c (%)	5.9 ± 0.2	5.9 ± 0.2	6.3 ± 0.4	6.0 ± 0.2	5.8 ± 0.2	5.7 ± 0.2	5.8 ± 0.1	5.7 ± 0.1	ns	ns	ns
Fasting C-peptide (pM)	1344.9 ± 157.6	1354.9 ± 130.9	898.4 ± 74.7	929.2 ± 81.7	881.5 ± 81.1	895.4 ± 117.7	1081.6 ± 99.3	1046.8 ± 123.3	ns	ns	ns
Fasting insulin (mU/l)	9.9 ± 3.3	14.1 ± 2.1	14.5 ± 2.4	15.2 ± 2.4	13.6 ± 2.4	12.6 ± 1.6	**18.1 ± 5.0**	13.6 ± 3.4*	**0.005**	ns	ns
Fasting glucose (mg/dl)	108.7 ± 5.6	106.7 ± 5.8	110.9 ± 7.5	111.4 ± 9.5	97.5 ± 5.0	95.3 ± 4.4	103.9 ± 4.3	99.7 ± 4.0	ns	ns	ns
HOMA IR	4.3 ± 0.7	4.8 ± 0.7	3.3 ± 0.4	3.6 ± 0.5	3.0 ± 0.5	3.0 ± 0.3	**4.7 ± 0.9**	4.2 ± 1.0*	0.07	ns	ns
HOMA ISI	0.3 ± 0.1	0.3 ± 0.0	0.4 ± 0.1	0.4 ± 0.1	0.5 ± 0.1	0.4 ± 0.0	**0.3 ± 0.0**	0.4 ± 0.1*	ns	0.09	ns
Matsuda	3.6 ± 0.5	3.2 ± 0.9	3.1 ± 0.6	3.5 ± 0.6	3.4 ± 0.7	3.3 ± 0.4	**2.5 ± 0.4**	3.0 ± 0.5*	ns	ns	ns
DPP-IV activity (mUI/ml)	17.7 ± 1.8	17.3 ± 2.0	20.5 ± 1.7	21.1 ± 1.8	17.4 ± 1.3	16.5 ± 1.3*	**18.1 ± 1.3**	16.4 ± 1.5*	ns	ns	ns

Values are means ± SEM (MAL ↓PA = placebo group: *n* = 12; MAL ↑PA = placebo group with increased PA: *n* = 19, INU ↓PA = prebiotic group: *n* = 16 and INU ↑PA = inulin group with increased PA: *n* = 14). A Wilcoxon matched pair was performed for evaluating differences within groups (3 months versus baseline). Significant results for Wilcoxon test are indicated in bold, *p < 0.05, †q < 0.05 (FDR correction). Mixed models were performed to evaluate the effects of different variables between groups (inulin treatment and PA variables as fixed effects and patients and hospitals as random effects). *BMI* Body mass index, *AST* Aspartate aminotransferase, *ALT* Alanine aminotransferase, *gGT* Gamma-glutamyl transferase, *LDL* Low-density lipoprotein, *HDL* High-density lipoprotein, *HbA1c* Hemoglobin A1c, *DPP-IV* Dipeptidyl-peptidase IV, *AUC* Area under the curve during an oral glucose tolerance test, *HOMA-IR* Homeostasis model assessment of insulin resistance, *HOMA ISI* HOMA insulin sensitivity index

Using questionnaires, we evaluated the appearance and persistence of gastrointestinal symptoms. Few changes were observed for nausea and reflux (Additional file: Fig. S1A-B). Rumbling, cramps, bloating and flatulence significantly increased in inulin group, as assessed by the area under the curve (Additional file: Fig. S1C-G). Flatulence decreased with time and PA reduced bloating and cramps in the inulin group (Additional file: Fig. 1D-G). Collectively, these data suggest that increasing voluntary PA during prebiotic supplementation improves the weight loss, metabolic parameters, and gastrointestinal tolerance in obese individuals.

### PA enhances the bifidogenic effects of inulin in obese individuals

**Fig. 2:**
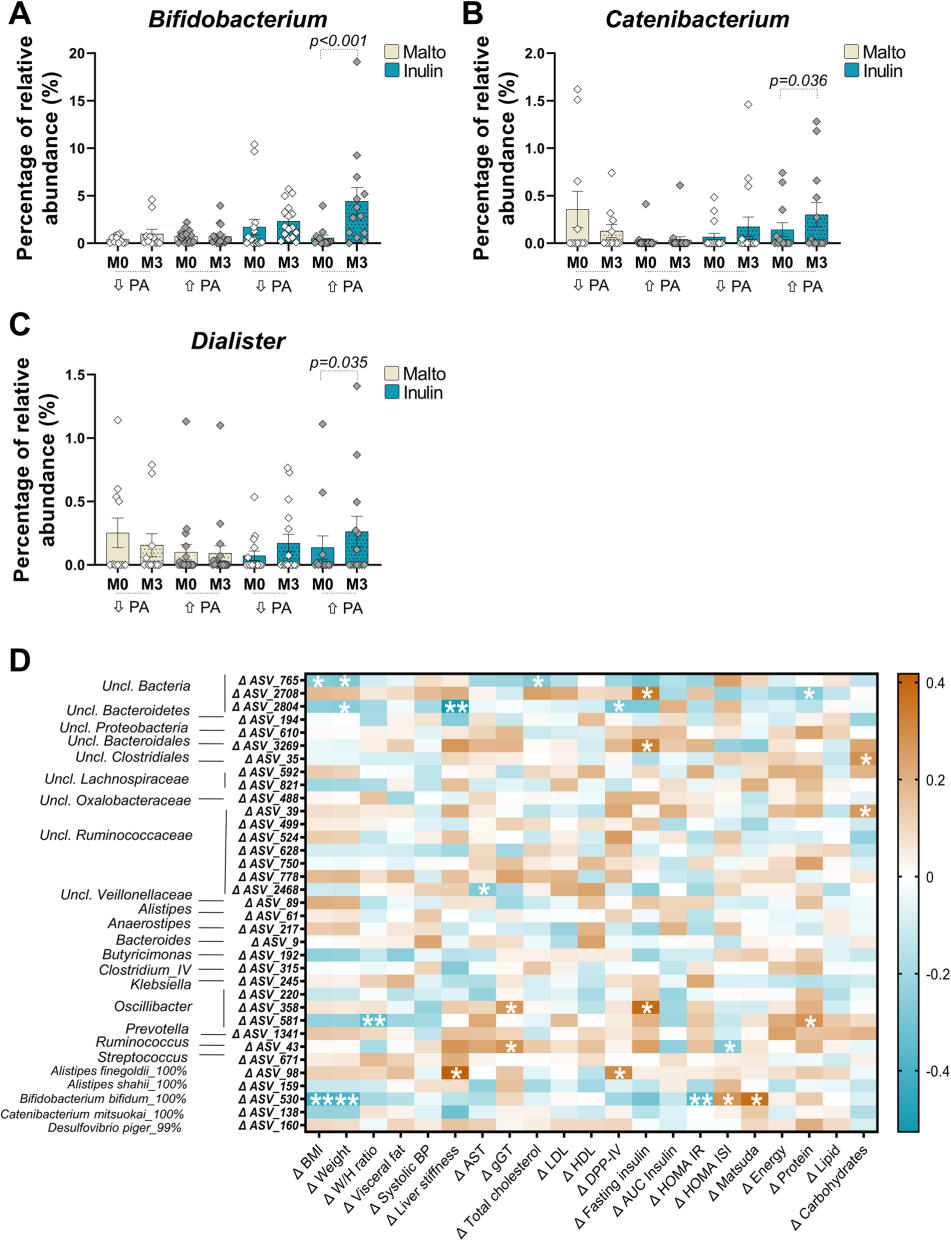
Impact of PA and inulin supplementation on the gut microbiota composition in obese individuals. **A**–**C** Percentage of relative abundance of *Bifidobacterium*, *Catenibacterium*, and *Dialister* genera. **D** Heatmap of Spearman’s correlations between amplicon sequence variants (ASV) significantly modified by inulin treatment and the most significant metabolic changes observed with inulin during intervention. *p < 0,05 and **p < 0,01

The overall gut microbiota composition was not impacted by PA or inulin, as shown by the different α-diversity indices related to bacterial richness or evenness (Additional file: Fig. S2A-F). Discriminant analysis based on a dissimilarity matrix using the Bray-Curtis index did not show clear specific clusters between the different groups, before and after intervention (Additional file: Fig. S2G). At baseline, only two genera were differently expressed between the groups (Additional file: Table 2). However, univariate analysis revealed significant changes at phyla, family, and genus levels (Table 2). We observed a marked increase in Actinobacteria, Bifidobacteriaceae, and *Bifidobacterium* in the inulin with increased PA group*. Bifidobacterium* genus tended to increase with inulin supplementation (*p = 0.06*), but this effect was much stronger in combination with PA (*p* and *q < 0.05*, Fig. 2A, Table 2). A significant increase of relative *Catenibacterium* and *Dialister* abundance was observed only in the inulin with increased PA group (Fig. 2B, C, Table 2). We also observed an increase of *Anaerostipes* genus upon inulin, independently of PA changes, as well as a decrease of *Clostridium sensu stricto* in two groups of participants who increased their PA (Table 2)*.*

**Table 2:** Taxa regulated by inulin and/or PA in the gut microbiota of obese individuals

Mean ± SEM (within groups: Wilcoxon matched pair tests)													*P*-value
	MAL ↓PA			MAL - ↑PA			INU ↓PA			INU - ↑PA			
	Baseline	Month 3	Change	Baseline	Month 3	Change	Baseline	Month 3	Change	Baseline	Month 3	Change	
**Phyla**													
Actinobacteria	0.987 ± 0.199	2.057 ± 0.947	1.07 ± 0.834^ab^	1.433 ± 0.258	1.333 ± 0.302	− 0.1 ± 0.329^b^	2.394 ± 0.896	3.096 ± 0.593	0.702 ± 1.097^ab^	**1.458 ± 0.421**	5.234 ± 1.641 *†	3.776 ± 1.385^a^	**0.003†**
**Family**													
Bifidobacteriaceae	0.397 ± 0.115	0.982 ± 0.489	0.585 ± 0.431^ab^	0.725 ± 0.138	0.713 ± 0.227	− 0.012 ± 0.257^a^	1.699 ± 0.83	2.33 ± 0.463	0.63 ± 1.034^bc^	**0.565 ± 0.299**	4.423 ± 1.445 *†	3.858 ± 1.462^c^	**< 0.001†**
Clostridiaceae	0.011 ± 0.007	0.063 ± 0.054	0.052 ± 0.056	**0.909 ± 0.775**	0.1 ± 0.042 *	− 0.809 ± 0.748	0.077 ± 0.039	0.056 ± 0.026	− 0.021 ± 0.041	**0.066 ± 0.032**	0.017 ± 0.015 *	− 0.048 ± 0.025	ns
Erysipelotrichaceae	0.935 ± 0.17	0.879 ± 0.189	− 0.056 ± 0.238	0.346 ± 0.112	0.337 ± 0.073	− 0.009 ± 0.103	**0.671 ± 0.172**	1.063 ± 0.259 *	0.392 ± 0.135	1.036 ± 0.331	1.717 ± 0.525	0.681 ± 0.316	**0.044**
Lachnospiraceae	19.591 ± 2.381	18.97 ± 2.436	− 0.621 ± 2.025	**19.775 ± 1.652**	15.669 ± 1.659 *	− 4.106 ± 1.452	18.613 ± 1.949	16.92 ± 1.491	− 1.693 ± 1.486	19.985 ± 1.725	17.657 ± 1.498	− 2.328 ± 1.852	ns
Oxalobacteraceae	0.066 ± 0.024	0.053 ± 0.017	− 0.013 ± 0.017^a^	0.035 ± 0.014	0.052 ± 0.019	0.017 ± 0.01^b^	0.034 ± 0.014	0.023 ± 0.009	− 0.011 ± 0.006^a^	0.03 ± 0.014	0.01 ± 0.006	− 0.019 ± 0.014^a^	**0.005**
Peptostreptococcaceae	**0.106 ± 0.041**	0.045 ± 0.028 *	− 0.061 ± 0.026	0.07 ± 0.022	0.609 ± 0.528	0.539 ± 0.531	0.103 ± 0.029	0.098 ± 0.049	− 0.005 ± 0.055	0.11 ± 0.051	0.051 ± 0.014	− 0.06 ± 0.045	ns
Ruminococcaceae	23.136 ± 1.695	24.655 ± 2.56	1.518 ± 2.27	**19.294 ± 1.486**	21.755 ± 1.866 *	2.461 ± 1.1	15.651 ± 1.605	18.408 ± 2.371	2.758 ± 1.828	15.978 ± 2.223	16.965 ± 1.876	0.986 ± 1.618	ns
Veillonellaceae	0.441 ± 0.141	0.311 ± 0.122	− 0.13 ± 0.113	1.526 ± 0.936	1.147 ± 0.478	− 0.38 ± 1.016	1.994 ± 0.921	2.267 ± 0.882	0.273 ± 0.681	**1.544 ± 0.841**	1.599 ± 0.63 *	0.055 ± 0.3	ns
**Genus**													
*Anaerostipes*	0.139 ± 0.038	0.145 ± 0.042	0.007 ± 0.04	0.165 ± 0.056	0.126 ± 0.025	− 0.039 ± 0.065	**0.126 ± 0.069**	0.504 ± 0.198 *	0.378 ± 0.146	**0.129 ± 0.047**	0.639 ± 0.27 *	0.51 ± 0.246	**0.046**
*Bifidobacterium*	0.397 ± 0.115	0.982 ± 0.489	0.585 ± 0.431^ab^	0.725 ± 0.138	0.712 ± 0.227	− 0.013 ± 0.257^a^	1.699 ± 0.83	2.328 ± 0.462	0.629 ± 1.034^bc^	**0.565 ± 0.299**	4.423 ± 1.445 *†	3.859 ± 1.462^c^	**< 0.001†**
*Catenibacterium*	0.357 ± 0.189	0.129 ± 0.07	− 0.228 ± 0.128^a^	0.023 ± 0.022	0.035 ± 0.032	0.012 ± 0.01^ab^	0.066 ± 0.037	0.174 ± 0.101	0.108 ± 0.073^b^	**0.142 ± 0.073**	0.301 ± 0.129 *	0.159 ± 0.061^b^	**< 0.001†**
*Clostridium sensu stricto*	0.011 ± 0.007	0.063 ± 0.054	0.052 ± 0.056	**0.909 ± 0.775**	0.1 ± 0.042 *	− 0.809 ± 0.748	0.077 ± 0.039	0.056 ± 0.026	− 0.021 ± 0.041	**0.066 ± 0.032**	0.017 ± 0.015 *	− 0.048 ± 0.025	ns
*Clostridium IV*	1.923 ± 0.561	1.136 ± 0.342	− 0.787 ± 0.359^b^	**1.061 ± 0.217**	1.505 ± 0.306 *	0.444 ± 0.18^a^	0.586 ± 0.098	1.139 ± 0.4	0.554 ± 0.396^ab^	1.194 ± 0.297	1.014 ± 0.324	− 0.18 ± 0.393^b^	**0.006**
*Dialister*	0.253 ± 0.117	0.156 ± 0.091	− 0.097 ± 0.102	0.1 ± 0.061	0.092 ± 0.059	− 0.008 ± 0.016	0.072 ± 0.036	0.172 ± 0.069	0.099 ± 0.058	**0.137 ± 0.092**	0.263 ± 0.12 *	0.126 ± 0.046	ns
*Escherichia Shigella*	0.519 ± 0.299 *	**0.237 ± 0.144**	− 0.282 ± 0.163	0.472 ± 0.164	0.69 ± 0.409	0.218 ± 0.458	0.372 ± 0.155	0.979 ± 0.786	0.607 ± 0.82	0.708 ± 0.36	0.568 ± 0.293	− 0.14 ± 0.385	ns
*Holdemanella*	0.248 ± 0.079	0.287 ± 0.114	0.039 ± 0.089	**0.071 ± 0.028**	0.138 ± 0.057 *	0.067 ± 0.044	0.47 ± 0.173	0.749 ± 0.246	0.278 ± 0.123	0.734 ± 0.346	1.203 ± 0.5	0.469 ± 0.255	ns
*Odoribacter*	1.625 ± 0.382	1.26 ± 0.293	− 0.364 ± 0.257	**1.386 ± 0.151**	2.241 ± 0.535 *	0.856 ± 0.532	1.408 ± 0.223	1.264 ± 0.254	− 0.143 ± 0.208	1.609 ± 0.294	1.333 ± 0.27	− 0.276 ± 0.376	ns
*Roseburia*	3.311 ± 1.293	2.455 ± 0.525	− 0.856 ± 1.25	2.879 ± 0.574	2.305 ± 0.662	− 0.574 ± 0.683	**3.197 ± 0.636**	1.822 ± 0.631 *	− 1.375 ± 0.853	2.116 ± 0.619	1.151 ± 0.188	− 0.966 ± 0.583	ns
*Ruminococcus*	0.444 ± 0.188	1.448 ± 0.535	1.005 ± 0.587^a^	**0.989 ± 0.424**	1.527 ± 0.553 *	0.538 ± 0.383^a^	0.749 ± 0.268	1.159 ± 0.424	0.41 ± 0.237^a^	0.674 ± 0.217	0.272 ± 0.086	− 0.403 ± 0.194^b^	**0.019**

Values refer to the relative abundances of taxa significantly regulated during the intervention. A Wilcoxon matched pair was performed for evaluating differences within groups (3 months versus baseline). Significant results for Wilcoxon test are indicated in bold, *p < 0.05, †*q* < 0.05 (FDR correction). A Kruskal-Wallis test was performed for comparisons in the change of relative abundances during the protocol, between all groups. *p*-value are indicated when *p* < 0.05, †*q* < 0.05 (FDR correction). For between comparisons, a different letter was attributed when the variations between groups are significant. MAL ↓PA = placebo group: *n* = 11; MAL ↑PA = placebo group with increased PA: *n* = 19, INU ↓PA = prebiotic group: *n* = 16 and INU ↑PA = inulin group with increased PA: *n* = 13

We then performed an amplicon sequence variant (ASV) analysis and identified 35 ASV differently regulated between groups (Additional file: Table 3). Among these, specific ASV belonging to *Bifidobacterium* (ASV530) and *Anaerostipes* (ASV217) were increased in inulin with PA. We then performed correlation analyses between the significant changes observed for clinical outcomes and ASV (Fig. 2D). The change in ASV530 was negatively associated with changes in BMI, weight, and HOMA-IR and positively correlated with changes in HOMA-ISI and Matsuda indexes. ASV358 (*Oscillibacter*) positively correlated with gGT and fasting insulin, and ASV98 was positively associated with liver stiffness and DPP-IV activity. Other ASV, such as ASV765 and ASV2804 (that cannot be classified into known taxa), were negatively associated with BMI, weight, total cholesterol, or liver stiffness.

Among ASV belonging to *Bifidobacterium* genus (the main known inulin target), ASV24 (99.6% similarity with *B. faecale* on EZ biocloud database) was most increased in inulin plus PA group (Additional file: Table 4). ASV26 *(B. adolescentis*) and ASV530 (*B. bifidum*) also increased specifically in the inulin + PA group, whereas the increase of ASV362 (*B. angulatum)* was found in both inulin groups, independently of PA changes. ASV158 (*B. pseudocatenulatum)* tended to increase in the feces of participants receiving inulin and increasing PA. Taken together, these data strongly support that PA changes the fermentation profile and influences the growth of some bacteria targeted by inulin.

### Voluntary exercise, combined to inulin supplementation, enhances the gut transit and the cecal fermentation in high fat diet-fed mice

**Fig. 3:**
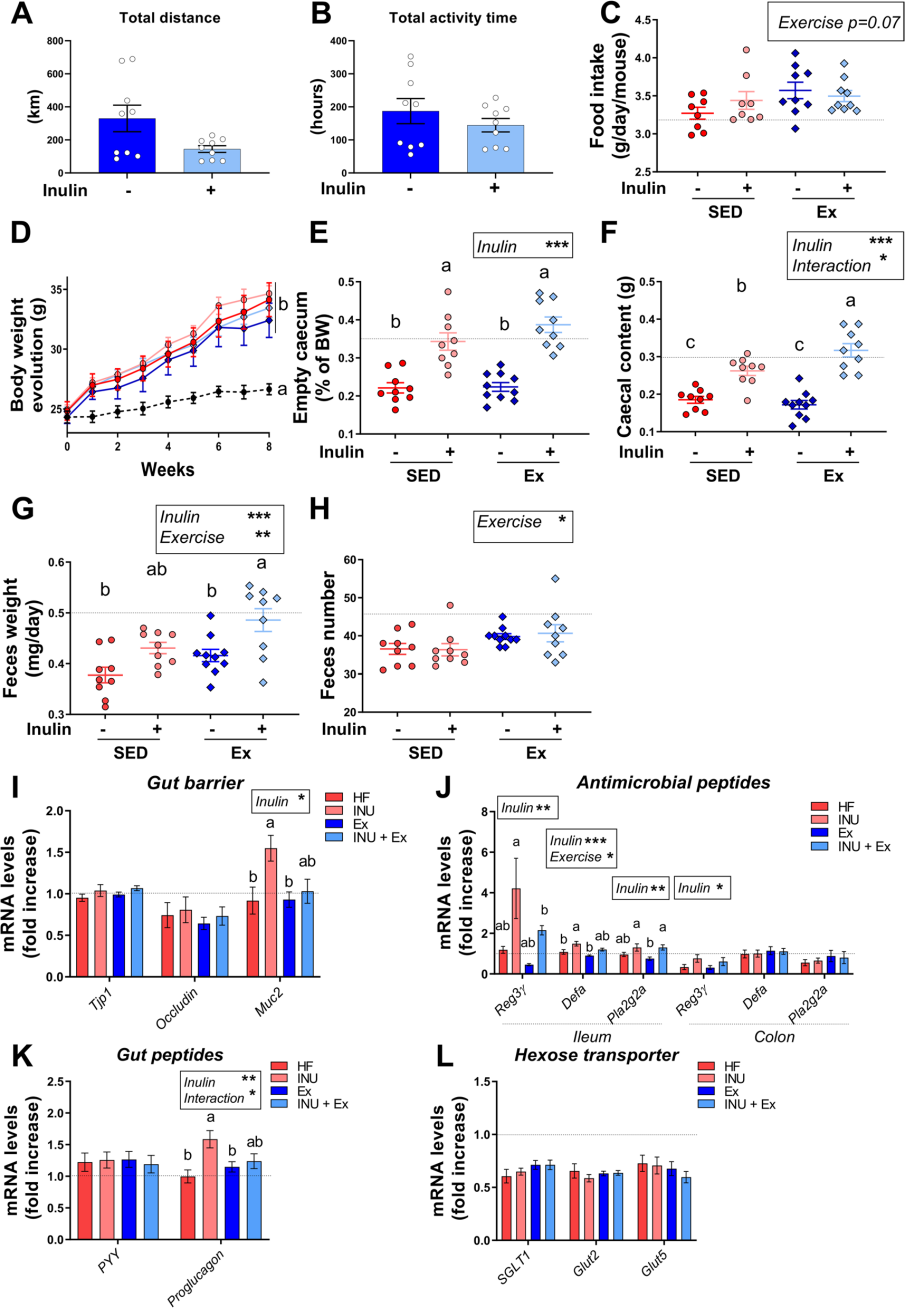
Impact of PA and inulin supplementation on gut transit and intestinal physiology. **A**, **B** Total distance and total activity time, performed by mice, are indicated in km and hours, respectively, in the two groups receiving a running wheel (Ex and INU+Ex groups). **C** Daily food intake in grams per mouse. **D** Body weight evolution in grams all along the experiment. **E**, **F** Cecal content and tissue weight. **G**, **H** Feces weight and number collected during a period of 24 h. **I** Gene expression of gut barrier markers measured by qPCR in the colon. **J** Gene expression for antimicrobial peptides markers measured by qPCR in both ileum and colon. **K** Gene expression measured by qPCR in the colon. **L** Gene expression for hexose transporter markers, measured by qPCR in the jejunum. For each panel, data are expressed as means ± SEM per group. The black dotted line represent the mean obtained for the control group fed with a standard diet. For the other four groups fed with a high-fat diet, a two-way ANOVA was performed and results for each variables (inulin, exercise or interaction) are shown in the box when significant. A Tukey post hoc test was also performed for comparison between groups and a different letter was attributed when the groups exhibit significant differences

To investigate the mechanisms by which prebiotic combined with voluntary exercise (Ex) can exert beneficial effects on host physiology and gut microbiota composition, we subjected mice to voluntary exercise (Ex) and inulin supplementation. Mice were fed a high-fat (HF) diet to induce obesity-related metabolic disorders. They were supplemented with 0.3 g inulin/day/mouse during 8 weeks with or without a voluntary running wheel placed in the cage. Daily Ex recording showed that the total distance run tended to be reduced upon inulin (*p* = 0.07 after log-transformation), whereas the time spent in the wheel was similar between the two groups of exercised mice (Fig. 3A, B). A tendency to increased food intake was observed with exercise (Fig. 3C), whereas no change in water intake and hence inulin intake was observed (data not shown). Mice on HF diet gained body weight in a similar way (Fig. 3D). As expected, inulin supplementation led to a significant increase in both cecal tissue and content (Fig. 3E, F). The increase of cecal content was greater in the group doing voluntary Ex, suggesting that Ex maximized the gut fermentation of inulin. 24-h feces weight was higher in the group receiving inulin and running wheel compared to other groups, and Ex increased feces number (Fig. 3G, H). These data suggest that Ex regulates the gut transit, a possible explanation for the increased cecal content when Ex is combined with inulin supplementation.

We next examined intestinal physiology. Inulin significantly increased colonic mRNA level of *Mucin2* (*Muc2*, a marker of mucopolysaccharide synthesis that is involved in gut barrier function), especially in sedentary mice (Fig. 3I). Inulin also increased the ileum mRNA levels of antimicrobial peptides *Regenerating islet-derived protein 3-gamma (Reg3ϒ)*, *α-defensin (Defa)*, and *Phospholipase A2 Group IIA (Pla2g2a)* (Fig. 3J). Colon *Reg3ϒ* mRNA expression was also increased by inulin. Ex did not affect these markers. Finally, we measured the colon mRNA expression of peptide YY (PYY) and proglucagon. PYY was similar between all HF groups whereas inulin significantly increased proglucagon, in particular in sedentary mice (Fig. 3K). We then analyzed glucose transporters in the jejunal portion, i.e., sodium glucose co-transporter 1 (*SGLT1*) and the glucose transporter 2 (*Glut2*) and *Glut5*. The hexose transporters decreased with HF diet compared to regular chow, but there were no differences between HF groups (Fig. 3L).

Altogether, Ex combined with inulin did not affect body weight gain in HF-fed mice. Interestingly, it did promote inulin-driven gut fermentation as indicated by the increased cecal content, an effect pointing to changes in the gut microbiota.

### Voluntary exercise modulates gut microbiota profile upon inulin treatment in HF-fed mice

**Fig. 4:**
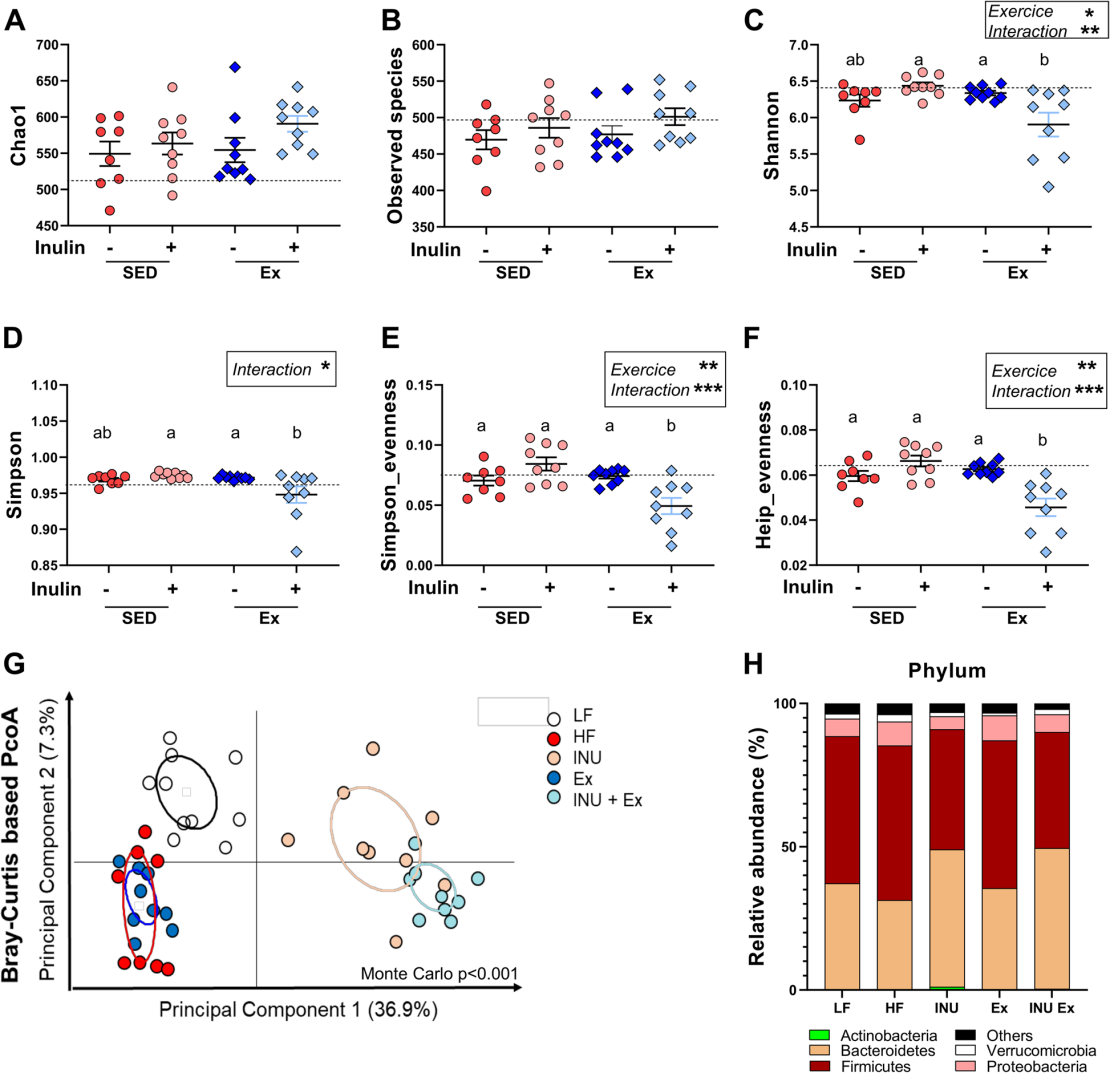
Effect of voluntary exercise and inulin supplementation on the gut microbiota composition in mice. Measure of alpha-diversity indexes: chao-1 (**A**), number of observed species (**B**), Shannon (**C**), Simpson (**D**), Simpson-evenness (**E**), and Heip-evenness (**F**). A two-way ANOVA was performed for evaluating the effects of inulin, exercise and the interaction between both variables (Inulin x exercise), between the four groups receiving a high-fat diet. The black dotted line represents the result obtained in the group of mice fed with a standard diet. Multiples comparisons were then assessed using Tukey’s post hoc test. A different letter was attributed to the groups when the variations between these groups are significant. **G** Principal coordinate analysis (PCoA) of the Bray-Curtis distance (β-diversity index), colored by group. **H** Barplots representing the mean of relative abundance of phyla in each group

Because Ex exacerbated the gut fermentation of inulin, we analyzed the mouse gut microbiota. Inulin significantly increased the number of total bacteria in the cecal content, and this effect was amplified when combined with Ex (Additional file: Fig. S3A). In the feces, the total bacteria level was higher at the fourth week in both inulin groups and it tended to be higher when combined with Ex at the end of the experiment (Additional file: Fig. S3B). We performed 16S rRNA sequencing of cecal content. Neither inulin nor exercise affected the bacterial richness indicators chao1 and the number of observed ASV (Fig. 4A, B). However, Ex decreased the indexes taking into consideration the evenness and we observed an interaction between inulin and Ex on the regulation of the indexes of Simpson, Shannon, Simpson evenness, and Heip evenness (Fig. 4C–F). Indeed, a reduction of these indicators was observed in the cecal content of mice receiving inulin and exposed to the running wheel. Principal coordinate analysis (PCoA) of the Bray-Curtis distance to visualize different clusters revealed a strong effect of inulin on the overall gut microbiota composition (ADONIS permanova, *p* < 0.001 for inulin effect, Fig. 4G). At the phyla level, univariate analysis indicated several significant changes between groups (Fig. 4H, Table 3). The increase of Actinobacteria by inulin was only significant in the sedentary group, and this was consistent with the higher levels of both Bifidobacteriaceae family and *Bifidobacterium* genus in the inulin group compared to inulin plus Ex (Table 3). Other bacterial regulations observed after inulin treatment differ between the sedentary and active groups. The decrease of *Oscillibacter* and *Alistipes* by inulin was more pronounced in mice with Ex (Table 3). Similarly, the increase of *Ruminococcus* upon inulin treatment was higher in active than in sedentary mice.

**Table 3:** Taxa regulated by inulin and/or PA in the gut microbiota of mice

	***Mean ± SEM***					***Kruskal-Wallis test***	
	***LF***	***HF***	***INU***	***Ex***	***INU+ Ex***	***p-value***	***q-value***
***Phylum***							
Firmicutes	51.31 ± 1.3^a^	53.888 ± 2.866^a^	41.956 ± 1.555^b^	51.511 ± 1.468^a^	40.511 ± 2.228^b^	< 0.001	**< 0.001**
Bacteroidetes	37.09 ± 1.357^ab^	31.25 ± 1.885b	47.9 ± 1.123 ac	35.378 ± 1.05b	49.1 ± 1.553c	< 0.001	**< 0.001**
Actinobacteria	0.091 ± 0.026^a^	0.072 ± 0.016^a^	1.054 ± 0.243^b^	0.112 ± 0.029^a^	0.334 ± 0.149^ab^	0.001	**0.002**
Proteobacteria	6.092 ± 0.751^ab^	8.386 ± 1.38^a^	4.502 ± 0.688^b^	8.763 ± 0.902^a^	6.182 ± 0.571^ab^	0.005	**0.008**
**Family**							
Lachnospiraceae	7.789 ± 0.935^a^	6.3 ± 0.812^ab^	5.081 ± 1.016^b^	6.221 ± 0.614^ab^	4.497 ± 0.623^ab^	0.024	**0.035**
Ruminococcaceae	27.91 ± 1.44^a^	28.65 ± 2.351^ab^	16.678 ± 1.5^bc^	28.867 ± 1.047^a^	9.791 ± 0.689^c^	< 0.001	**< 0.001**
Coriobacteriaceae	0.087 ± 0.025^ab^	0.063 ± 0.014^ab^	0.209 ± 0.071^a^	0.09 ± 0.025^ab^	0.056 ± 0.02^b^	0.017	0.029
Rikenellaceae	11.562 ± 1.723^ab^	11.866 ± 1.499^ab^	8.651 ± 1.26^ab^	13.822 ± 0.855^a^	5.727 ± 0.391^b^	0.002	**0.005**
Prevotellaceae	0.673 ± 0.131^ab^	0.651 ± 0.213^ab^	1.419 ± 0.348^ab^	0.39 ± 0.056^a^	1.492 ± 0.267^b^	0.003	**0.005**
Clostridiales_Incertae_Sedis_XIII	0.089 ± 0.011^a^	0.079 ± 0.009^ab^	0.038 ± 0.007^ab^	0.092 ± 0.017^a^	0.032 ± 0.005^b^	0.001	**0.003**
Bifidobacteriaceae	0 ± 0^a^	0 ± 0^a^	0.842 ± 0.195^b^	0.015 ± 0.007^ab^	0.276 ± 0.133^b^	< 0.001	**< 0.001**
Peptostreptococcaceae	0.262 ± 0.09^a^	0.313 ± 0.068^a^	0.002 ± 0.001^b^	0.468 ± 0.275^a^	0.002 ± 0.002^b^	< 0.001	**< 0.001**
Desulfovibrionaceae	0.263 ± 0.021^a^	0.342 ± 0.049^ab^	0.318 ± 0.075^ab^	0.594 ± 0.093^b^	0.168 ± 0.038^a^	0.002	**0.005**
***Genus***							
*Oscillibacter*	8.902 ± 0.597^ab^	10.54 ± 1.009^ab^	6.264 ± 0.794^bc^	10.742 ± 0.524^a^	2.768 ± 0.334^c^	< 0.001	**< 0.001**
*Clostridium_IV*	0.375 ± 0.054^a^	0.176 ± 0.013^ab^	0.118 ± 0.032^b^	0.163 ± 0.022^ab^	0.086 ± 0.016^b^	< 0.001	**< 0.001**
*Alistipes*	11.562 ± 1.723^ab^	11.853 ± 1.496^a^	8.634 ± 1.255^ab^	13.822 ± 0.855^a^	5.718 ± 0.392^b^	0.002	**0.003**
*Prevotella*	0.173 ± 0.04^a^	0.042 ± 0.019^b^	0.106 ± 0.025^ab^	0.054 ± 0.012^ab^	0.165 ± 0.028^a^	0.003	**0.005**
*Parabacteroides*	1.869 ± 0.402^a^	0.113 ± 0.031^b^	0.143 ± 0.032^b^	0.343 ± 0.11^b^	0.895 ± 0.285^ab^	< 0.001	**< 0.001**
*Ruminococcus*	0.017 ± 0.006^ab^	0.013 ± 0.005^a^	0.071 ± 0.032^ab^	0.018 ± 0.005^ab^	0.115 ± 0.044^b^	0.004	**0.006**
*Bifidobacterium*	0 ± 0^a^	0 ± 0^a^	0.842 ± 0.195^b^	0.015 ± 0.007^ab^	0.276 ± 0.133^b^	< 0.001	**< 0.001**
*Eisenbergiella*	0.112 ± 0.052^a^	0.008 ± 0.002^ab^	0.001 ± 0^b^	0.01 ± 0.002^ab^	0.004 ± 0.002^b^	< 0.001	**< 0.001**
*Clostridium_XlVb*	0.049 ± 0.025^a^	0.006 ± 0.002^ab^	0 ± 0^b^	0.063 ± 0.02^a^	0.001 ± 0.001^b^	< 0.001	**< 0.001**
*Macellibacteroides*	0.015 ± 0.003^ab^	0.002 ± 0.001^a^	0.002 ± 0.001^a^	0.005 ± 0.004^a^	0.048 ± 0.017^b^	< 0.001	**< 0.001**
*Romboutsia*	0.262 ± 0.09^a^	0.313 ± 0.068^a^	0.002 ± 0.001^b^	0.468 ± 0.275^a^	0.002 ± 0.002^b^	< 0.001	**< 0.001**
*Saccharibacteria_genera_incertae_sedis*	0.017 ± 0.006	0.011 ± 0.005	0.039 ± 0.011	0.011 ± 0.004	0.041 ± 0.012	0.024	**0.031**
*Bilophila*	0.263 ± 0.021^a^	0.342 ± 0.049^ab^	0.318 ± 0.075^ab^	0.594 ± 0.093^b^	0.168 ± 0.038^a^	0.002	**0.004**

The relative abundance of taxa significantly regulated by inulin or voluntary exercise in the cecal content of mice. Results are expressed as mean of relative abundance ± SEM. Significantly affected taxa by inulin or voluntary exercise were identified using a Kruskal-Wallis ANOVA performed in R. *p*-value was adjusted (*q*-value, significant if *q* < 0.05) to control for the false discovery rate (FDR correction) for multiple testing. *Ns* Not significant. Multiples comparisons were then assessed using Dunn’s post hoc test in R. A different letter was attributed to the groups when the variations between groups are significantly different

We then quantified by quantitative PCR the level of specific bacteria in cecal content. We confirmed the greater increase of *Bifidobacterium* by inulin in sedentary mice (Additional file: Fig. S3C). *Roseburia*, another inulin-degrader, was also increased by inulin but more so in active mice (Additional file: Fig. S3D). No change in *Akkermansia muciniphila* was observed between groups (Additional file: Fig. 3E). *Lactobacillus* significantly increased upon inulin only in mice performing voluntary exercise (Additional file: Fig. S3F). Altogether, these data highlight differential effects of inulin supplementation on gut microbiota composition in sedentary or active mice.

### Voluntary exercise, but not inulin, reduces serum and hepatic lipids

Even if no change on total body weight was seen between groups, Ex limited the gain of fat mass and loss of fat-free mass upon HF diet compared to sedentary mice (Additional file: Fig. S4A-B). This was due to reduced subcutaneous adipose tissue mass; the mass of other adipose tissues remained similar between groups (Additional file: Fig. S4C-F).

**Fig. 5:**
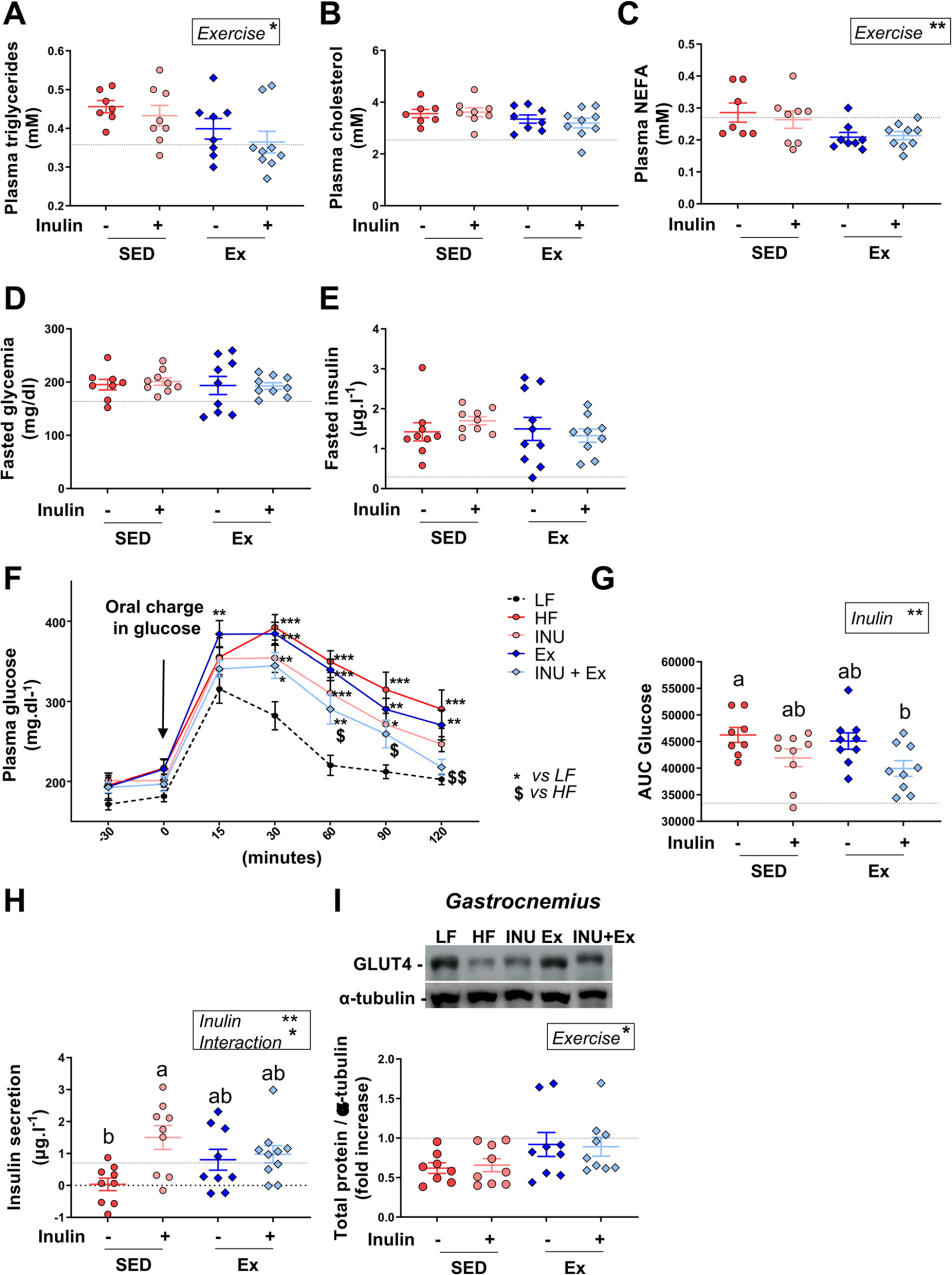
Voluntary exercise, combined with inulin supplementation, improved glucose tolerance in mice. **A**–**C** Fasted plasma triglycerides, cholesterol and NEFA. **D** Fasted plasma glucose (mg.dl^−1^). **E** Fasted plasma insulin (mg.L^−1^). **F** Glucose levels after an oral glucose load. **G** Area under the curve (AUC) of the glucose concentration evolution. **H** Insulin secretion corresponding to the difference of plasma insulin levels 30 min before and 15 min after the oral glucose load. **I**, **J** Immunoblotting and quantification of GLUT4 expression in *gastrocnemius* muscle, respectively. α-tubulin was used as protein loading control. Data are expressed as mean ± SEM. The black dotted line represents the mean obtained for the control group of mice fed with a standard diet. For the other four groups fed with a high-fat diet, a two-way ANOVA was performed, and significant results for each variable (inulin, exercise or interaction) are shown in the box. A Tukey post hoc test was then performed for comparison between groups and a different letter was attributed when the groups exhibit significant differences

Plasma triglycerides and non-esterified fatty acids (NEFA) significantly decreased in mice performing Ex, whereas cholesterol did not change (Fig. 5A–C). In the liver, Ex decreased total lipid accumulation whereas inulin had no effect (Additional file: Fig. S4G). Hepatic triglyceride and cholesterol content was not different between groups (Additional file: Fig. S4H-I). Ex tended to reduce the intramuscular lipid accumulation (Additional file: Fig. S4J-K). mRNA expression of fatty acid transport or oxidation genes was extensively not regulated by Ex in liver or muscle (Additional file: Fig. S5A-B). Only *peroxisome proliferator-activated receptor gamma coactivator 1-alpha* (*pgc-1α*) mRNA expression decreased in the liver of the exercised group, and it tended to decrease in both inulin groups (Additional file: Fig. S5A). *Sterol regulatory element-binding protein 1 (Srebp1c),* a driver of fatty acid synthesis, decreased upon inulin in the liver, but this effect was lost in combination with Ex. In the *gastrocnemius* muscle, inulin decreased the expression of fatty acid receptor *cluster of differentiation 36 (cd36)* and *long-chain fatty acid transport protein 4 (fatp4),* independently of Ex (Additional file: Fig. S5B).

### Voluntary exercise combined with inulin supplementation improves glucose tolerance in mice

At the seventh week of the experiment, we performed an oral glucose tolerance test (OGTT) in mice. Fasting glycemia and insulinemia were similar in all groups (Fig. 5D, E). The glycemia during the OGTT was reduced in mice receiving inulin and exposed to the running wheel, from 60 min on after the glucose gavage (Fig. 5F). At 120 min, the glycemia of mice on inulin plus Ex was comparable to that of chow-fed mice. The area under the curve for glucose showed that inulin and Ex each tended to improve glucose tolerance but that the combination had a significant effect (Fig. 5G). Inulin increased the early insulin peak measured 15 min after glucose load, and this effect was lowered when mice performed voluntary exercise (Fig. 5H). The expression of Glucose transporter 4 (GLUT4) protein was higher in skeletal muscle of exercised mice, whereas no regulation was observed with inulin (Fig. 5I). Taken together, these data show that combining inulin supplementation with exercise improves glucose tolerance in HF diet-fed mice, by targeting different organs and processes.

## Discussion

Previous studies proposed ITFs as interesting prebiotics with beneficial impact on host physiology. We have recently shown that internal and external factors influence the metabolic response to inulin in the context of obesity (intrinsic gut microbiota composition or metformin use) [12, 15]. Our current findings in obese study participants and in HF diet-fed mice suggest that increased PA enhances health outcomes of a nutritional intervention with inulin in obesity.

Previous studies performed in humans have suggested that the improvement of metabolic alterations and obesity upon inulin intervention in obesity varies between individuals. In our study, we demonstrated for the first time that several clinical improvements in obese individuals was observed mostly when PA was combined to a nutritional intervention with prebiotics supplementation, especially on the management of body weight and glucose homeostasis, but also on liver enzymes and total cholesterol. We also highlighted that the practice of PA during inulin supplementation may improve the gastrointestinal tolerance of high fermentable dietary fibers consumption. In parallel, a preclinical study corroborated the interest of inulin in mice to improve metabolic disorders, but as compared to existing data, it brought new insights into the mechanisms of action of inulin combined with PA.

Voluntary PA was not influenced by prebiotic intervention since we observed a similar change in PA upon the interventions both in obese people and HF-fed mice. This clearly suggests that inulin did not impact the motivation to perform exercise.

In the human cohort, we found a greater improvement of BMI when inulin was combined with PA. Waist/hip ratio and total cholesterol were reduced only in the group combining inulin with PA. This is in keeping with previous findings in rats fed a HF/high-sucrose diet, where the combination of treadmill training and a supplementation with fructans (a mix of inulin and oligofructose) prevented knee joint damage, usually observed in this rat model of obesity [33]. In this same study, training and prebiotics separately had beneficial effects on serum endotoxin and leptin levels and insulin sensitivity, whereas the combination of both reduced body fat and improved serum lipids. The same team recently showed that combining PA and prebiotic supplementation had no effect on glucose tolerance but improved body mass and LDL cholesterol when the treatment starts 12 weeks after the induction of obesity [34]. In our study, we observed decreased subcutaneous fat only by voluntary exercise in HF-fed mice. Obesity has multiple consequences, notably on the liver (with the development of metabolic dysfunction-associated fatty liver disease) [35]. In humans, we further found improved hepatic parameters with the combination of PA and inulin, such as a reduction of both AST and gGT. Interestingly, it has been recently demonstrated that exercise can regulate liver steatosis and stiffness in men with non-alcoholic fatty liver disease, independently of weight loss [36]. Here, we did not find a beneficial and global effect of PA on hepatic parameters, but we show that combination of PA and inulin is beneficial for the liver. In both humans and mice, we observed improved glucose tolerance and insulin sensitivity. The similar expression of hexose transporters in mouse jejunum suggests that diminished glucose absorption probably does not mediate the improved glucose tolerance.

It has been previously demonstrated that exercise can modulate gut microbiota composition in rodents [23, 24, 37–40]. Six weeks of treadmill running modified gut microbiota in normal and diabetic mice, but changes differed according to the metabolic status of mice [22]. In our mouse model, the index of Bray-Curtis distance shows that PA did not much alter overall gut microbiota composition, compared to the prebiotic-induced changes. Univariate analyses neither pointed to differences for taxa levels between HF and PA groups. This negligible impact of PA on gut microbiota is consistent with Lamoureux et al. who demonstrated that neither voluntary nor forced exercise contributed to microbiome changes [41]. The authors discussed that these results contradicting previous reports of significant impact of exercise on gut microbiota in animals and humans [20, 40, 42, 43] could be due to dietary or housing differences. Our data demonstrate that the impact of PA on gut microbiota depends on the diet as highlighted by the greater effects of PA combined with prebiotic supplementation compared to prebiotics alone. The effects of PA on gut microbiota and metabolic parameters are bigger when the diet contains prebiotic, compared to a HF diet poor in dietary fibers. Thus, PA can change gut transit and fermentation profiles and, therefore, diet plays a crucial role in mediating exercise effects on the gut microbiota. This can explain the lesser impact of PA in mice fed a diet poor in fibers, compared to inulin-supplemented mice. Recently, Liu et al. demonstrated that exercise induces a modest but distinguishable effect on the gut microbiota in metformin-naïve prediabetic individuals [25]. The response to high-intensity training was heterogenous in prediabetic subjects, associated with changes in insulin sensitivity and glucose metabolism. The microbiome of responders was associated with higher carbohydrate fermentation whereas that from non-responders produced metabolically detrimental compounds. Interestingly, fecal material transfer from responder individuals into antibiotic-pretreated mice mimicked the effect of exercise on glucose homeostasis. This provides support that fermentation features are important for mediating the effect of PA on metabolism.

In our study, we hypothesized that PA modifies gut bacterial fermentation and therefore influences the response to prebiotics. Indeed, we found a higher cecal content in inulin plus PA mice versus inulin-treated mice. Exercise can alter transit time and therefore regulate the substrate availability in the colon. Consistent with this, in HF-fed mice PA increased feces number and weight in the group receiving inulin. Unexpectedly, we observed in the obese cohort that gastrointestinal symptoms caused by inulin intake were reduced with PA. It is well established that gastrointestinal symptoms can appear during acute strenuous exercise [44]. In our cohort, the low- and moderate-intensity exercise conferred protective effects on the gastrointestinal tract with reduced cramps and bloating. PA may thus be considered in high dose prebiotic dietary interventions.

We examined whether combining PA with inulin changed the fermentation profile in obese individuals. Indeed, the stimulation of *Bifidobacterium* growth by inulin was more important when participants increased their PA during the protocol. Curiously, such a higher increase in *Bifidobacterium* genus was not found in HF-fed mice. Consistently, it has been previously demonstrated that inulin did not regulate all *Bifidobacterium* strains in the same way [45]. Since human and mice exhibit different strains of *Bifidobacterium*, this can explain the different bifidogenic effects induced by exercise combined with inulin supplementation in mice model versus human. Some other bacteria can be differently regulated by the prebiotic, according to PA. For instance, in mice, inulin decreased *Oscillibacter* and *Alistipes* more so when mice exercised. These bacterial regulations induced by ITF intake have been previously shown in a model of HF diet supplemented with gluten in mice [46]. However, it is interesting to observe that these ITF effects can be amplified with voluntary exercise. Inulin increased *Prevotella*, especially in combination with voluntary exercise. Some studies reported an increased in *Prevotella* abundance with dietary fibers in mice [46, 47]. An elegant study in humans reported that the improvement of glucose metabolism by a dietary fiber (barley kernel) is associated with an induction of *Prevotella* [48]. Consistent with this finding, we found improved glucose tolerance in mice on inulin plus PA in which we detected the higher increase of *Prevotella*. Our study highlighted that combining physical activity with a nutritional intervention based on prebiotic supplementation really improved clinical and metabolic parameters observed during obesity. However, the limitation if this exploratory work is that the study is not initially designed for evaluating the impact of PA on prebiotic supplementation since no advices were given to the participants in order to increase their PA. Well-powered studies are thus needed to evaluate the potential beneficial effects of inulin with PA on the metabolic outcomes in participants who follow a specific program of PA during the intervention in a larger cohort. In addition, the cohort included obese subjects with various associated metabolic alterations and diseases (hypertension, dyslipidemia, type 2 diabetes…). Due to number of participants within each group, it was not possible to evaluate and consider the effects of all these variables in this subcohort. Future investigations should focus on the effect of PA associated with prebiotic supplementation in each specific condition to evaluate the impact of combining these two strategies on specific metabolic condition. Finally, another limitation is the fact that we could not predict which bacterial functions and/or metabolites is involved in the metabolic improvements induced by PA combined to inulin supplementation. This can be considered as a perspective of our work to investigate in specific obesity-associated metabolic alterations.

## Conclusions

We highlight, in obese individuals and rodents, improvements in metabolic parameters and gastrointestinal tolerance by inulin when this supplementation was combined with voluntary PA. These beneficial effects could be explained by a difference in the gut fermentation profile of fermentable fibers. This study pinpoints the importance of diet for mediating beneficial effects of exercise in metabolic disorders. We believe that combining PA with dietary interventions including fermentable fibers with prebiotic properties will optimize outcomes in interventions in obese individuals.

## Supplementary Information

**Additional file 1: Figure S1*.*** Evaluation of gastrointestinal tolerance upon inulin supplementation, according to PA practice, in obese individuals. Score for gastrointestinal symptoms including nausea (A), reflux (B), rumbling (C), cramp (D), flatulence (E) and bloating (F), and area under the curve calculated for each symptoms (G). (*n*=9 for maltodextrin, *n*=15 for maltodextrin with increased PA, *n*=14 for inulin and *n*=10 for inulin with increased PA). Mixed-effects with repeated measures analysis were performed (inulin and PA variables as fixed effects, patients and hospitals as randomized effects). A post hoc test was then assessed for multiples comparisons. **Figure S2.** Impact of PA and inulin supplementation on the gut microbiota composition in obese individuals. Differences between the end (month 3, M3) and baseline (month 0, M0) for the measures of alpha-diversity indexes: chao-1 (A), number of observed species (B), Shannon (C), Simpson (D), Simpson-evenness (E) and Heip-evenness (F), (*n*=12 for maltodextrin, *n*=19 for maltodextrin with increased PA, *n*=16 for inulin and *n*=14 for inulin with increased PA). (G) Principal coordinates analysis (PCoA) of the Bray-Curtis distance (β-diversity index), colored by group. **Figure S3.** Specific bacteria analyzed by qPCR in DNA extracted from cecal content of mice. Total bacteria level in the cecal content (A) and feces (B) at baseline, week4 and week8. Levels of (C) *Bifidobacterium* spp. (D) *Roseburia* spp. (E) *Akkermansia muciniphila* and (F) *Lactobacillus* spp quantified in the cecal content of mice. For each panel, a dotted line represent the amount of bacteria measured in the cecal content of mice fed with a standard diet. A two-way ANOVA was performed for evaluating the effects of inulin, exercise and the interaction (Inulin x exercise) between the four groups receiving the high-fat diet. When significant, the result for two-way ANOVA in indicated in a box. Multiples comparisons were then assessed using Tukey’s post hoc test. A different letter was attributed to the groups when the variations between these groups are significant. **Figure S4.** Effects of PA and inulin supplementation on adiposity. (A-B) Evolution of fat-free and fat mass percentages during the protocol performed in mice. (C-F) Percentage of subcutaneous, epididymal, brown and visceral adipose tissues versus total body weight. (G-I) Liver lipids content, triglycerides and cholesterol levels. (J-K) Intramuscular lipids content and triglycerides level in the *gastrocnemius* muscle. For each analysis, data are expressed as mean ± SEM per group. The black dotted line represent the mean obtained for the control group fed with a standard diet. For the other four groups fed with a high-fat diet, a two-way ANOVA was performed and significant results for each variables (inulin, exercise or interaction) were shown in the box. A Tukey post hoc test was then performed for comparison between groups and a different letter was attributed when the groups exhibit significant differences. **Figure S5.** Effects of PA and inulin supplementation on hepatic and muscle gene expression related to metabolism. Hepatic (A) or muscle (B) gene expression measured by qPCR. Data are expressed as mean ± SEM. The black dotted line represents the mean obtained for the control group of mice fed with a standard diet. For the other four groups fed with a high-fat diet, a two-way ANOVA was performed and significant results for each variables (inulin, exercise or interaction) are shown in the box. A Tukey post hoc test was then performed for comparison between groups and a different letter was attributed when the groups exhibit significant differences.

**Additional file 2: Table 1.** Physical activity performed by participants. **Table 2.** Baseline characteristics of participants. **Table 3.** Differences between M3 and baseline for ASV significantly regulated between groups. **Table 4.** Regulation of Bifidobacterium ASV between month 3 and baseline.

**Additional file 3: Gels.** A/ Original gels with the samples in order. On the left: original gels with the molecular weight marker. On the right: images used for the quantification. LF: low-fat diet group (*n*=10); HF: high-fat diet group (*n*=8); I=inulin group (*n*=9); Ex= exercise group (*n*=9); ExI= exercise + inulin group (*n*=9); SD= the same sample from Ex group that has been charged on every gels for normalization. C+= positive control. B/ Original gels with Ponceau S staining.

**Additional file 4.** Additional information for methods.

**Additional file 5.** CONSORT 2010 checklist of information to include when reporting a randomised trial*.

**Additional file 6.** The ARRIVE Essential 10: Compliance Questionnaire.

**Additional file 7.** Flow diagram (adapted from CONSORT 2010 flow diagram).

## Data Availability

The raw sequencing data is deposited into the Sequence Read Archive (SRA) of NCBI (http://www.ncbi.nlm.nih.gov/sra) under BioProject (please contact author for data requests). Original and uncropped gels are provided in the additional file.

## Abbreviations

ALT: Alanine aminotransferase
AST: Aspartate aminotransferase
ASV: Amplicon sequence variant
BMI: Body mass index
Cd36: Cluster of differentiation 36
Defa: α-Defensin
DPP-IV: Dipeptidyl-peptidase IV
Ex: Exercise
Fatp4: Fatty acid transport protein 4
gGT: Gamma-glutamyl transferase
Glut: Glucose transporter
HbA1c: Hemoglobin A1c
HDL: High-density lipoprotein
HF: High-fat
HOMA IR: Homeostasis model assessment of insulin resistance
HOMA ISI: HOMA insulin sensitivity index
INU: Inulin
IPAQ: International Physical Activity Questionnaire
ITF: Inulin-type fructans
LDL: Low-density lipoprotein
MAFLD: Metabolic associated fatty liver disease
Muc2: Mucin 2
OGTT: Oral glucose tolerance test
PA: Physical activity
PCoA: Principal coordinates analysis
Pgc1α: Peroxisome proliferator-activated receptor gamma coactivator 1-alpha
Pla2g2a: Phospholipase A2 group IIA
PYY: Peptide YY
Reg3ϒ: Regenerating islet-derived protein 3-gamma
SGLT1: Sodium glucose co-transporter 1
Srebp1c: Sterol regulatory element-binding protein 1c
