# Supplementary material for: Physical activity enhances the improvement of body mass index and metabolism by inulin: a multicenter randomized placebo-controlled trial performed in obese individuals
Source: BMC Med. 2022 Mar 30;20:110. doi: 10.1186/s12916-022-02299-z (PMC8966292; doi:10.1186/s12916-022-02299-z)
Supplement: Supplementary file 1 — Additional file 1: Figure S1. Evaluation of gastrointestinal tolerance upon inulin supplementation, according to PA practice, in obese individuals. Score for gastrointestinal symptoms including nausea (A), reflux (B), rumbling (C), cramp (D), flatulence (E) and bloating (F), and area under the curve calculated for each symptoms (G). (n=9 for maltodextrin, n=15 for maltodextrin with increased PA, n=14 for inulin and n=10 for inulin with increased PA). Mixed-effects with repeated measures analysis were performed (inulin and PA variables as fixed effects, patients and hospitals as randomized effects). A post hoc test was then assessed for multiples comparisons. Figure S2. Impact of PA and inulin supplementation on the gut microbiota composition in obese individuals. Differences between the end (month 3, M3) and baseline (month 0, M0) for the measures of alpha-diversity indexes: chao-1 (A), number of observed species (B), Shannon (C), Simpson (D), Simpson-evenness (E) and Heip-evenness (F), (n=12 for maltodextrin, n=19 for maltodextrin with increased PA, n=16 for inulin and n=14 for inulin with increased PA). (G) Principal coordinates analysis (PCoA) of the Bray-Curtis distance (β-diversity index), colored by group. Figure S3. Specific bacteria analyzed by qPCR in DNA extracted from cecal content of mice. Total bacteria level in the cecal content (A) and feces (B) at baseline, week4 and week8. Levels of (C) Bifidobacterium spp. (D) Roseburia spp. (E) Akkermansia muciniphila and (F) Lactobacillus spp quantified in the cecal content of mice. For each panel, a dotted line represent the amount of bacteria measured in the cecal content of mice fed with a standard diet. A two-way ANOVA was performed for evaluating the effects of inulin, exercise and the interaction (Inulin x exercise) between the four groups receiving the high-fat diet. When significant, the result for two-way ANOVA in indicated in a box. Multiples comparisons were then assessed using Tukey’s post hoc test. A [file 12916_2022_2299_MOESM1_ESM.pdf]

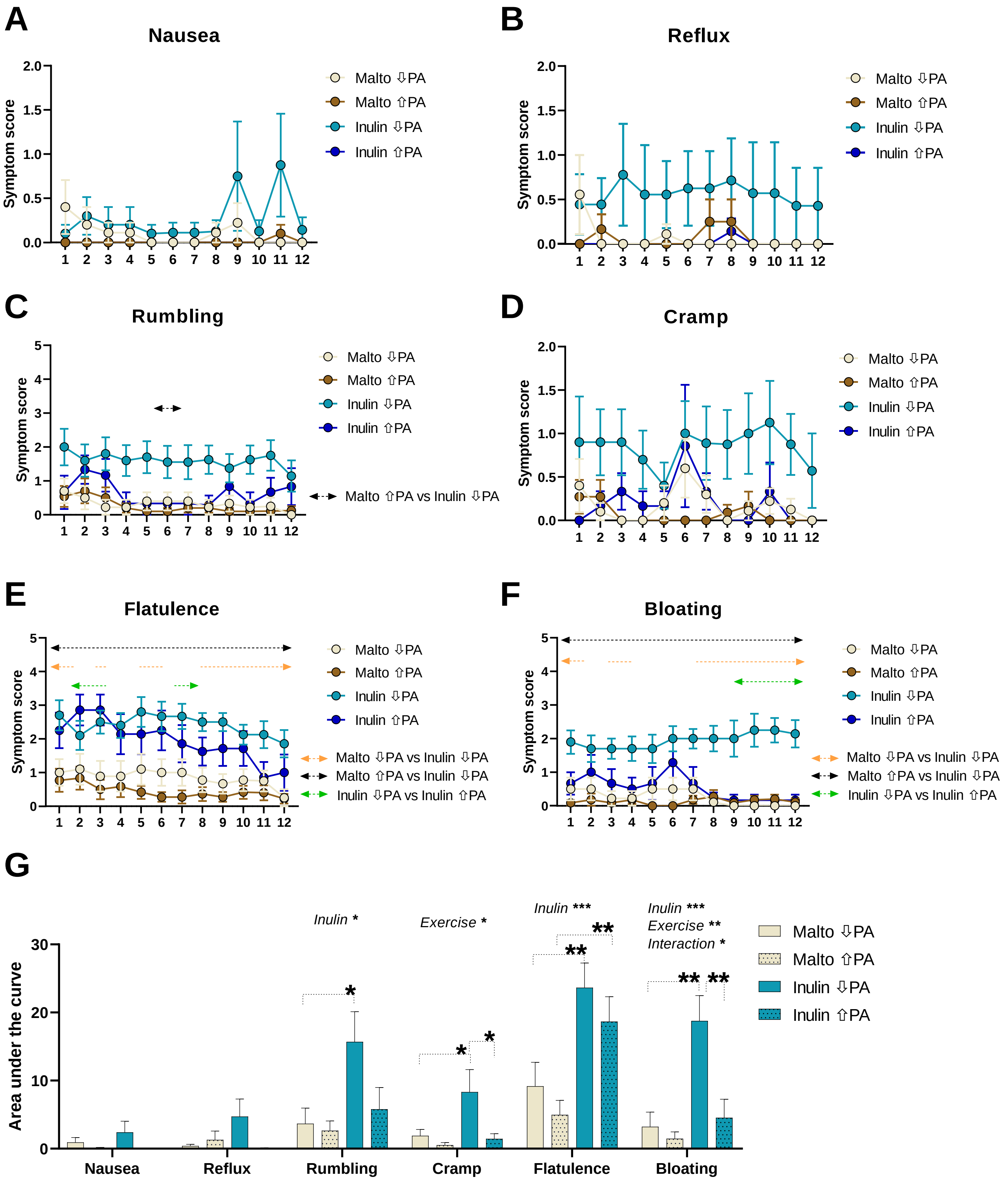

Additional file: Figure S1

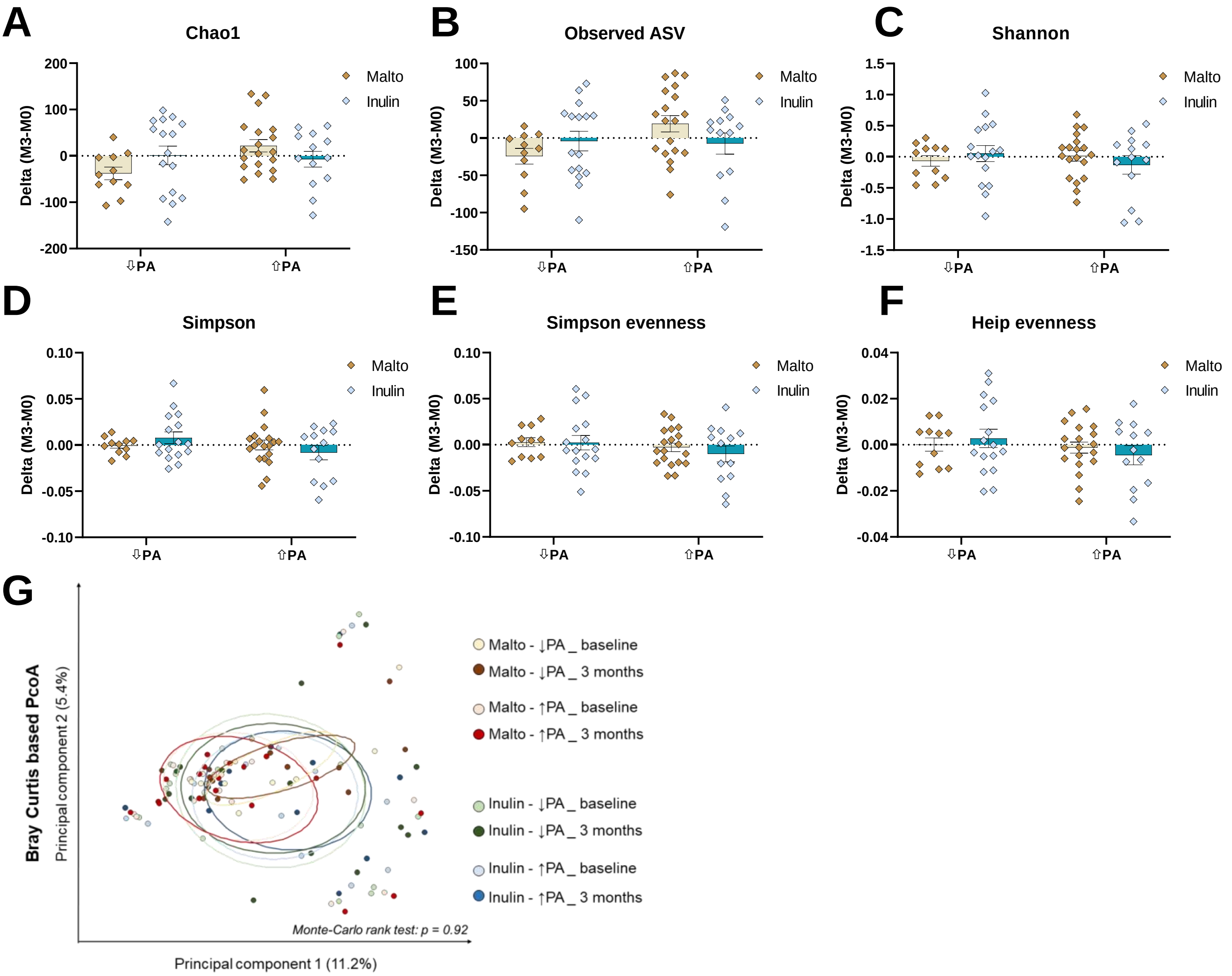

Additional file: Figure S2

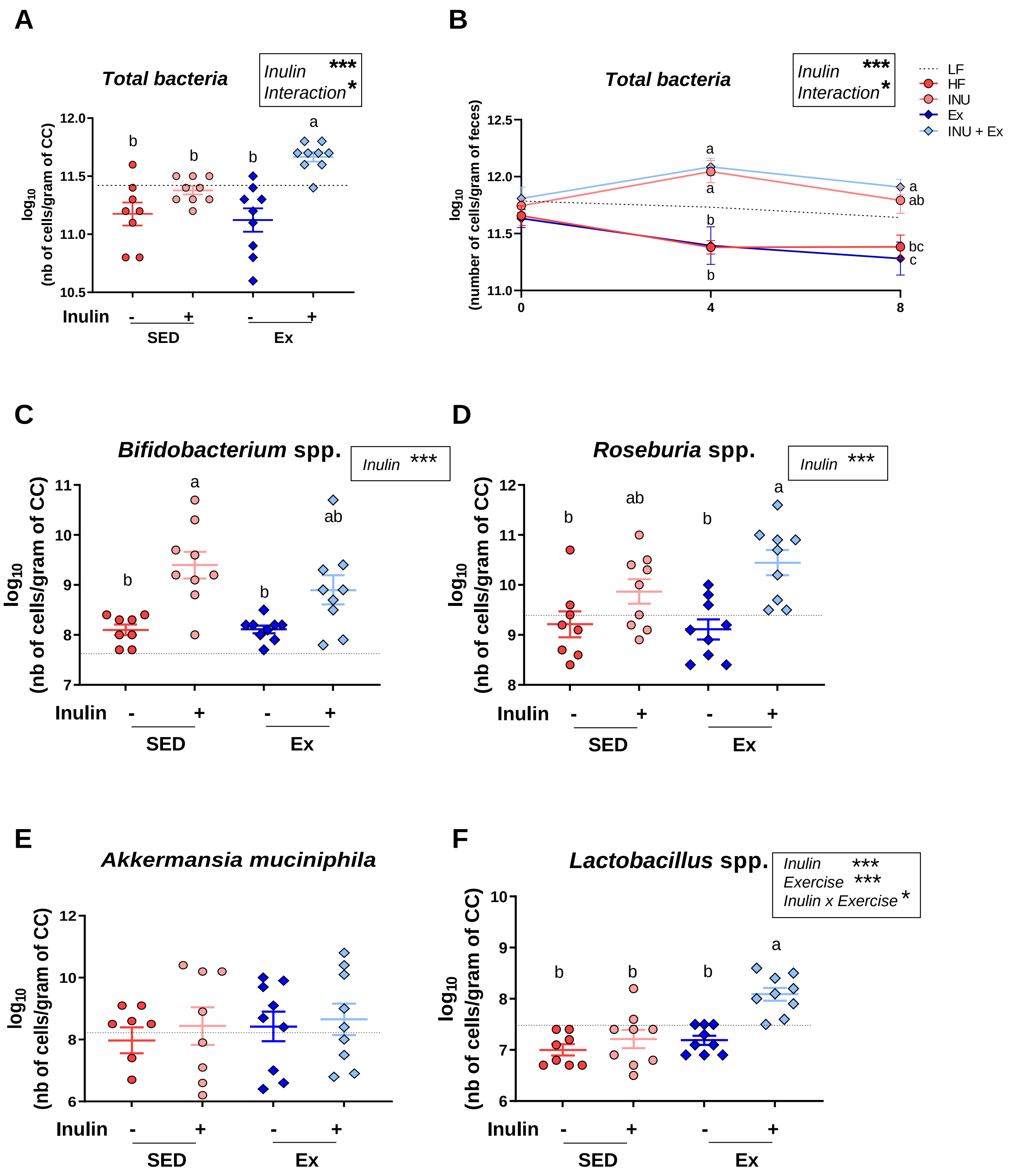

Additional file: Figure S3

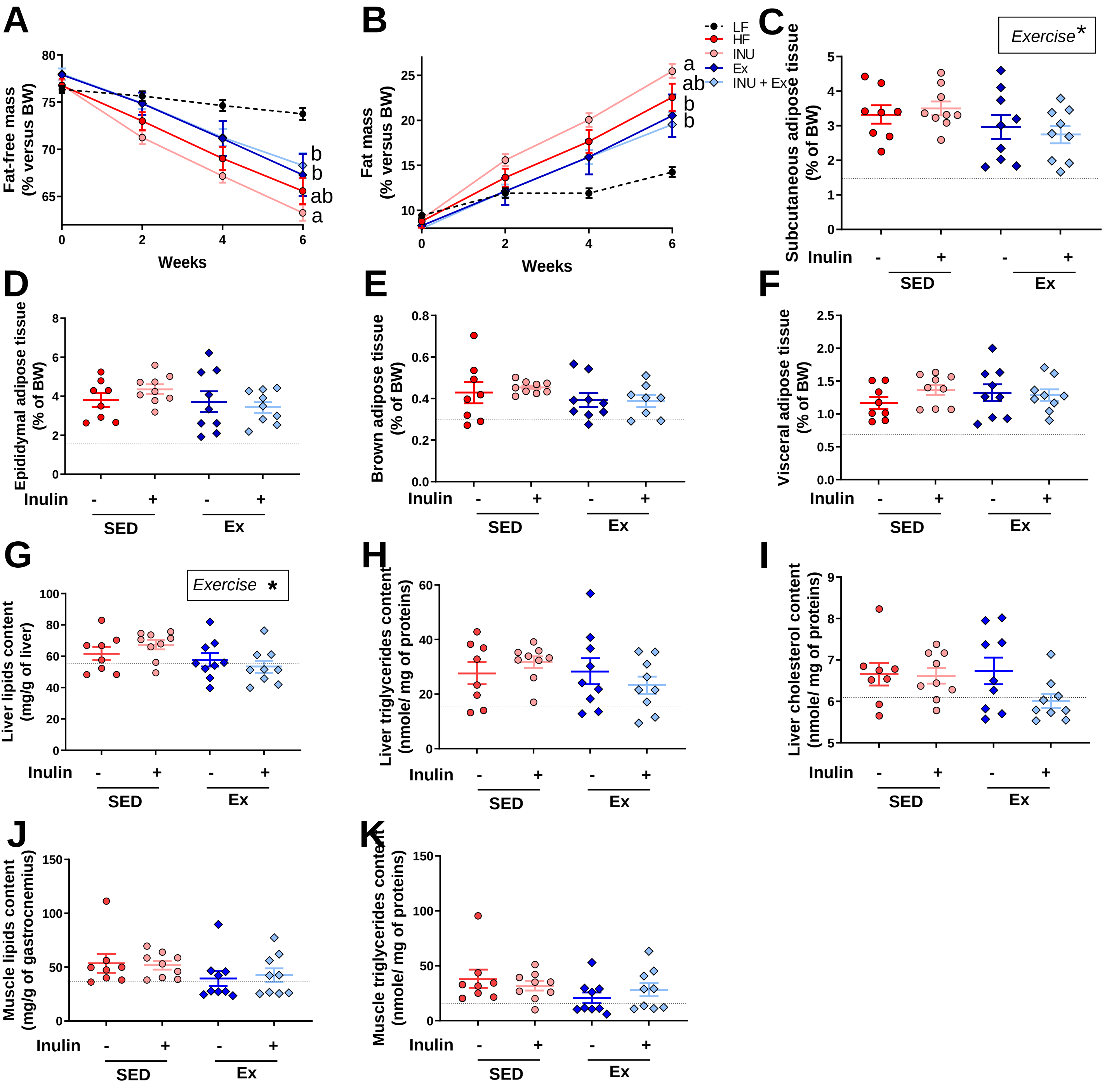

Additional file: Figure S4

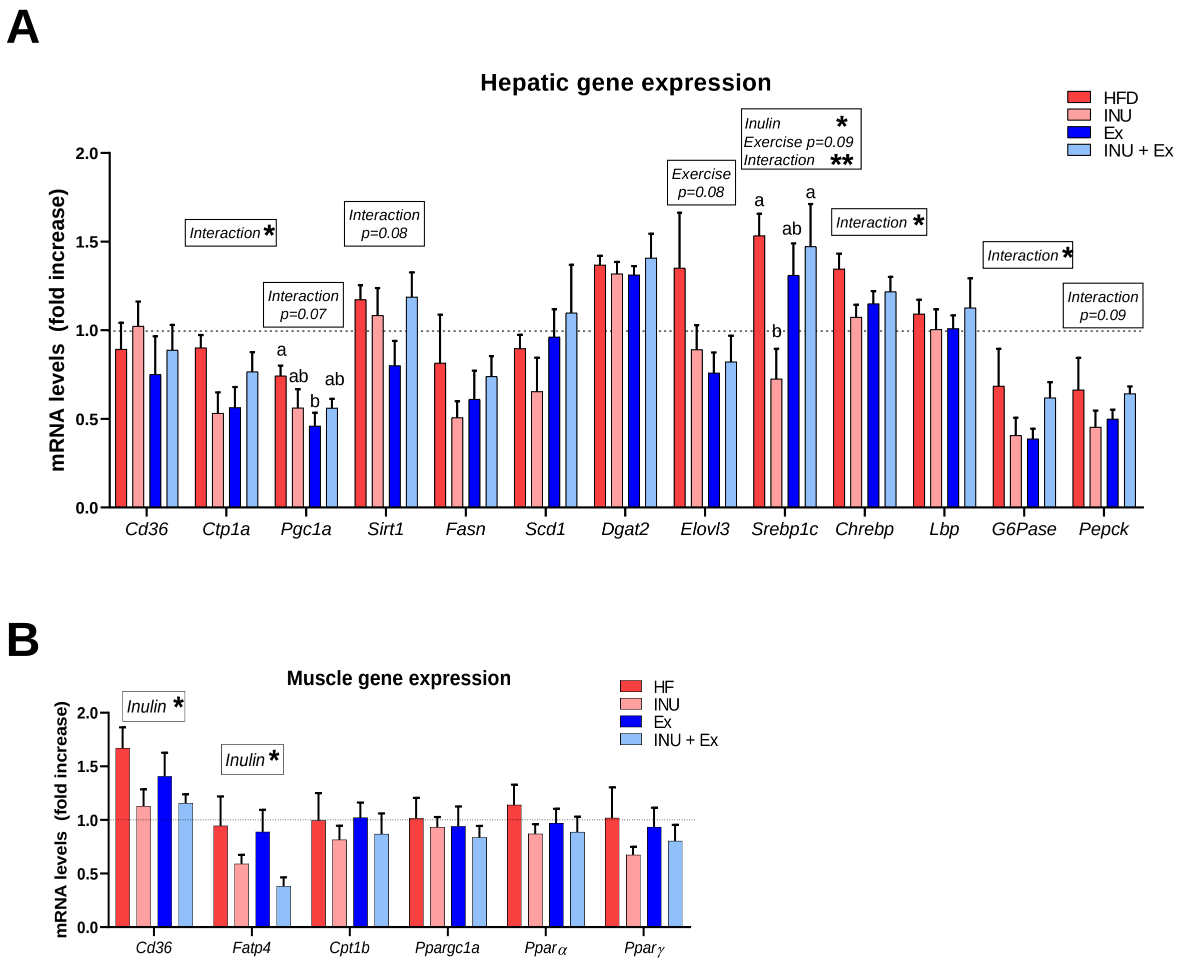

Additional file: Figure S5
