# Supplementary figures and images for: Physical activity enhances the improvement of body mass index and metabolism by inulin: a multicenter randomized placebo-controlled trial performed in obese individuals
Source: BMC Med. 2022 Mar 30;20:110. doi: 10.1186/s12916-022-02299-z (PMC8966292; doi:10.1186/s12916-022-02299-z)

A/Western blot: GLUT4 antibody

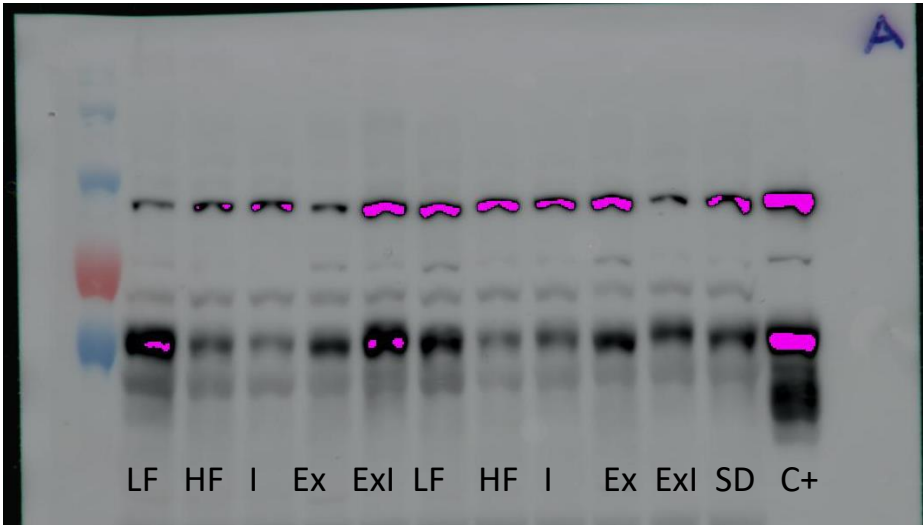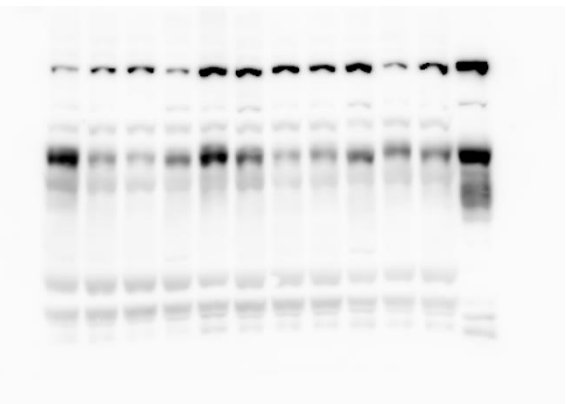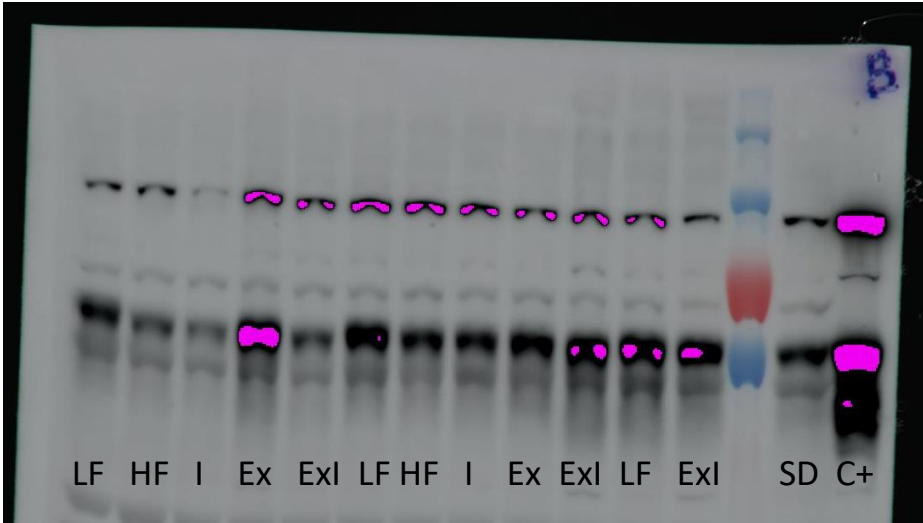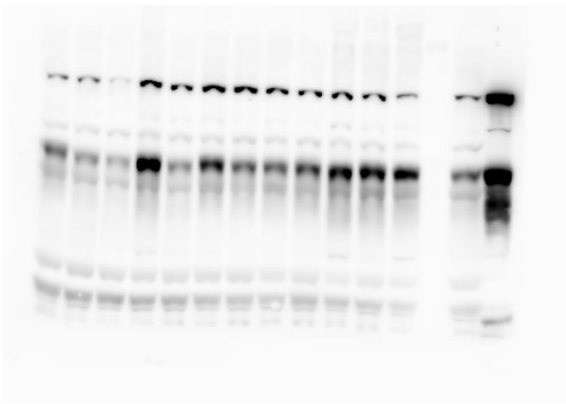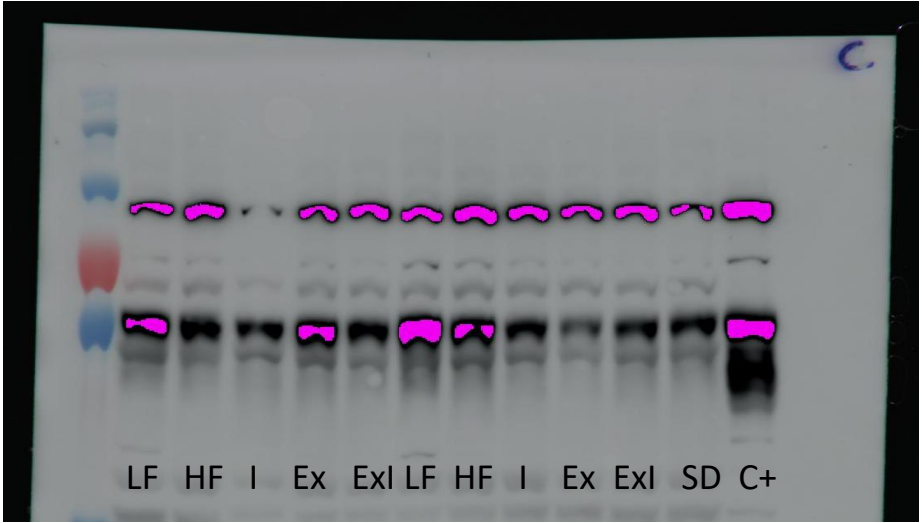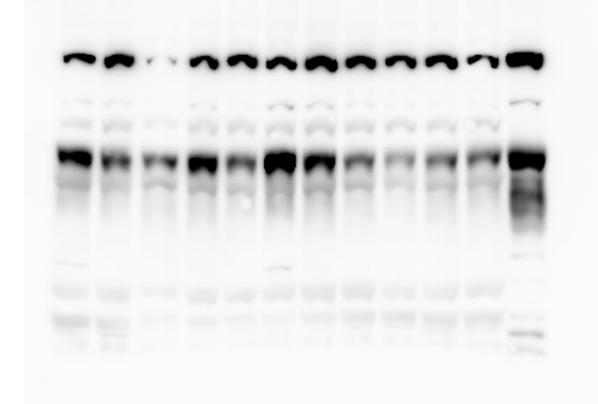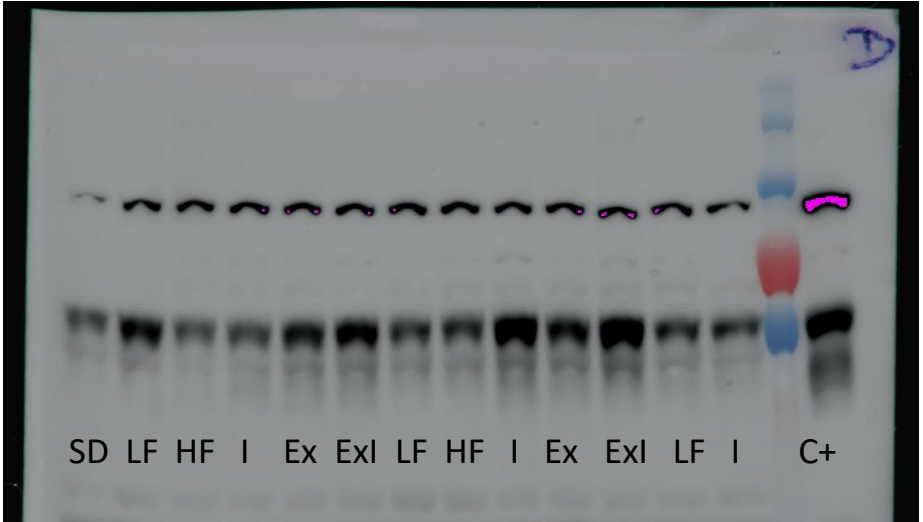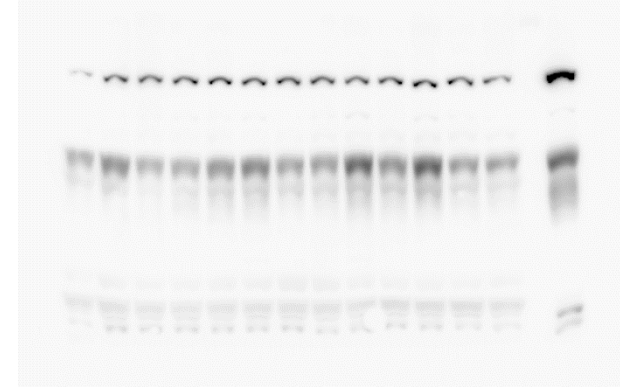

## B/ Staining with Ponceau S solution

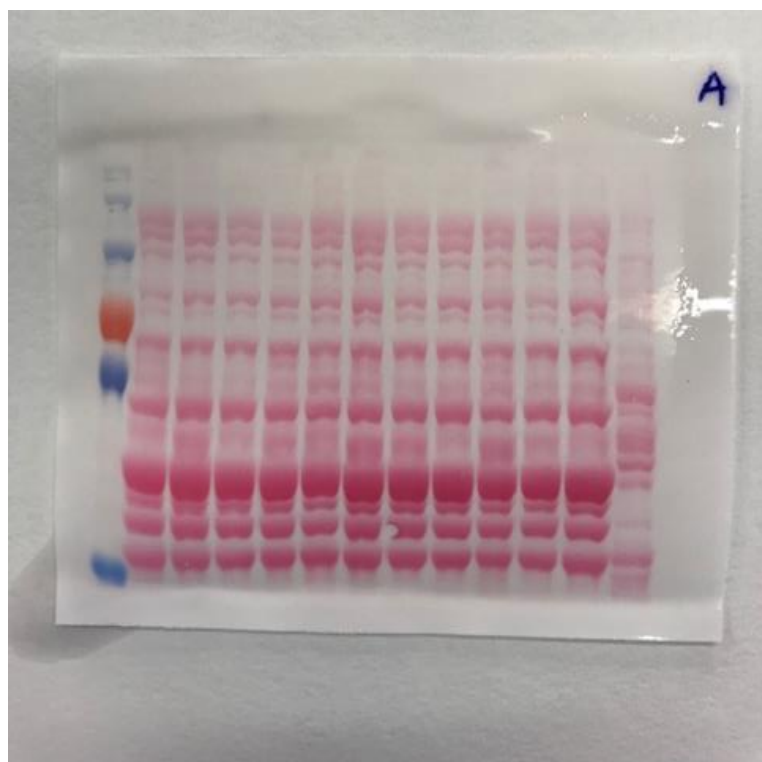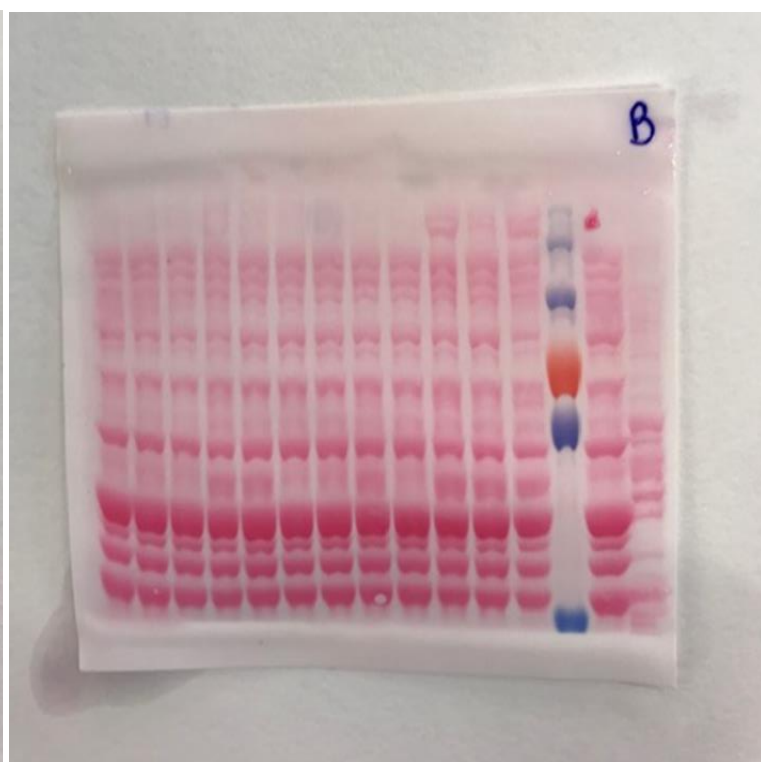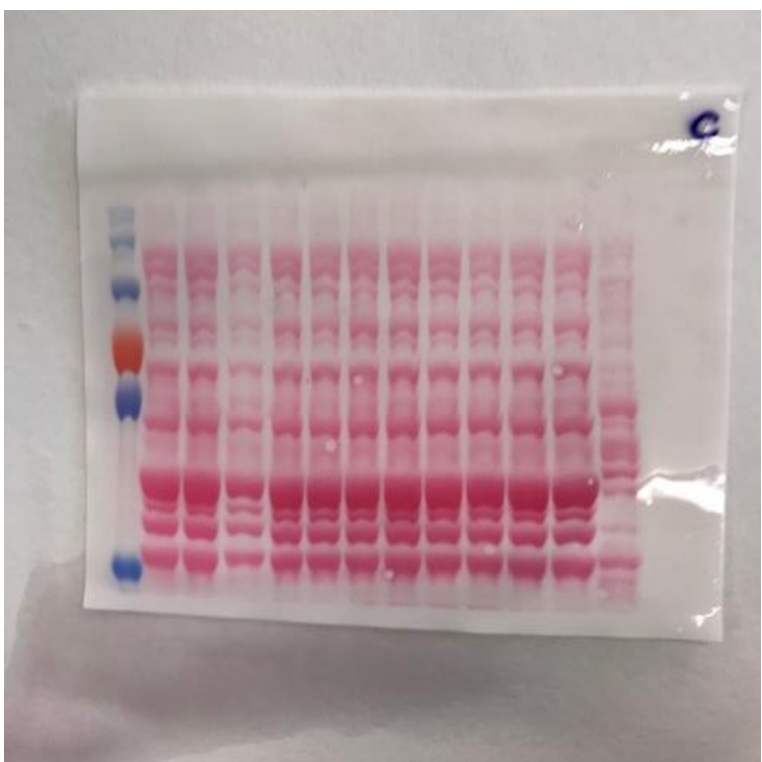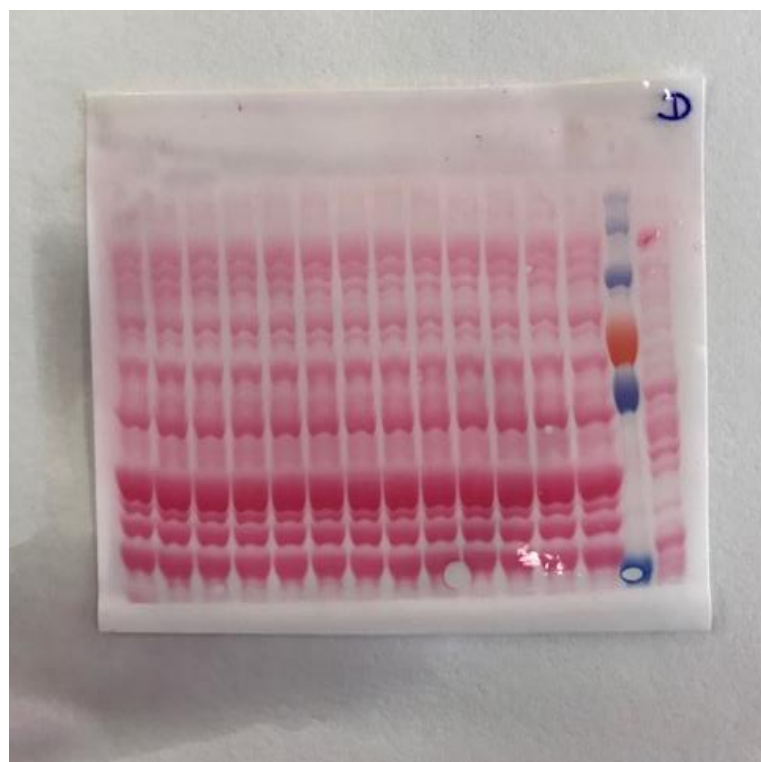

Supplement: Supplementary file 3 — Additional file 3: Gels. A/ Original gels with the samples in order. On the left: original gels with the molecular weight marker. On the right: images used for the quantification. LF: low-fat diet group (n=10); HF: high-fat diet group (n=8); I=inulin group (n=9); Ex= exercise group (n=9); ExI= exercise + inulin group (n=9); SD= the same sample from Ex group that has been charged on every gels for normalization. C+= positive control. B/ Original gels with Ponceau S staining. [file 12916_2022_2299_MOESM3_ESM.pdf]

Flow diagram (adapted from CONSORT 2010 flow diagram)

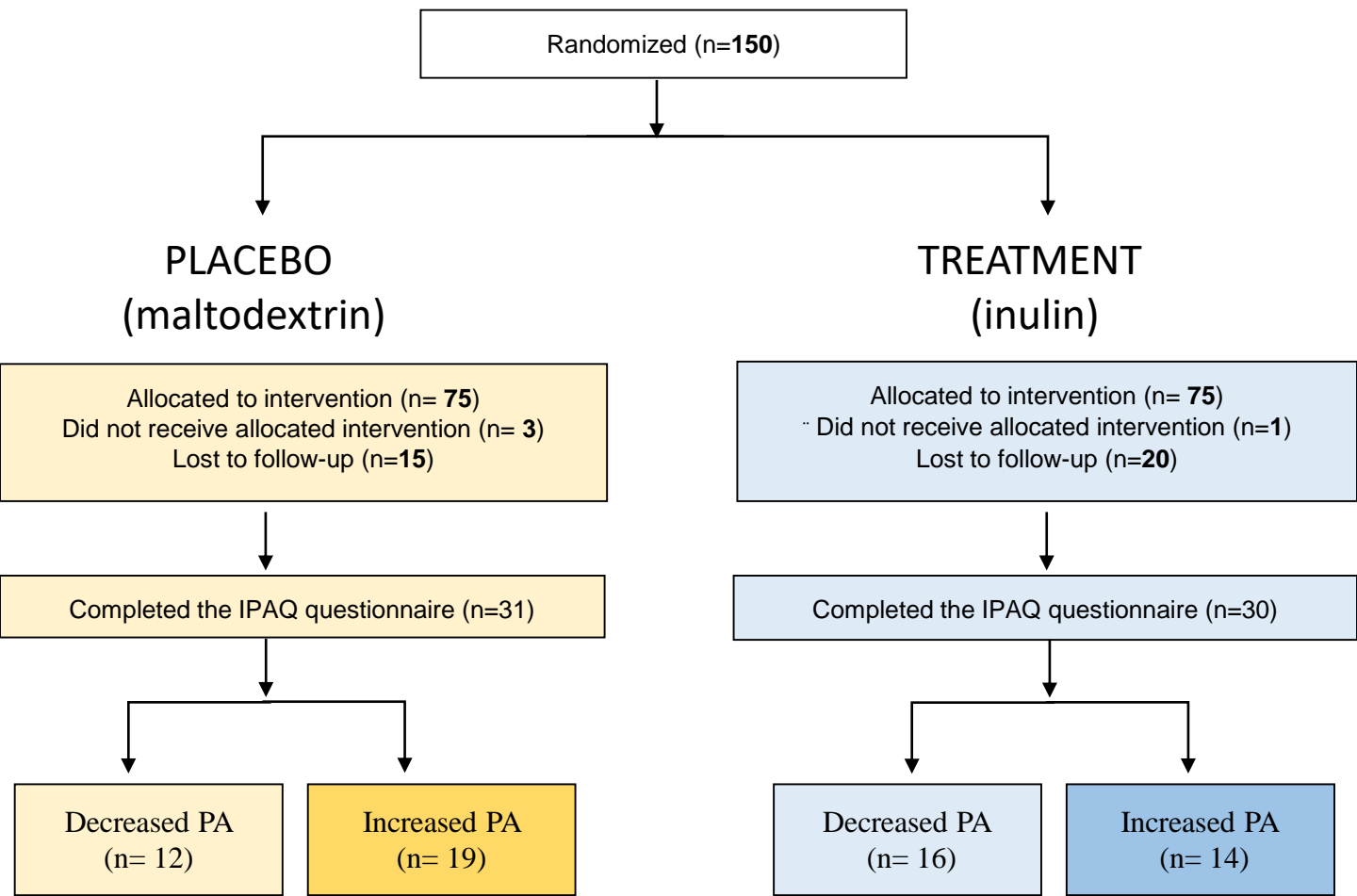

Supplement: Supplementary file 7 — Additional file 7. Flow diagram (adapted from CONSORT 2010 flow diagram). [file 12916_2022_2299_MOESM7_ESM.pdf]
