# Supplementary material for: Physical activity enhances the improvement of body mass index and metabolism by inulin: a multicenter randomized placebo-controlled trial performed in obese individuals
Source: BMC Med. 2022 Mar 30;20:110. doi: 10.1186/s12916-022-02299-z (PMC8966292; doi:10.1186/s12916-022-02299-z)
Supplement: Supplementary file 4 — Additional file 4. Additional information for methods. [file 12916_2022_2299_MOESM4_ESM.docx]

**ADDITIONAL INFORMATIONS**

**Physical activity enhances the improvement of body mass index and metabolism by inulin:  a multicenter randomized placebo-controlled trial performed in obese individuals.**

**Authors**

Julie Rodriguez^1^, Audrey M. Neyrinck^1^, Maxime Van Kerckhoven^1^, Marco A. Gianfrancesco^2^, Edith Renguet^3^, Luc Bertrand^3^, Patrice D. Cani^1,4^, Nicolas Lanthier^5^, Miriam Cnop^6^, Nicolas Paquot^2^, Jean-Paul Thissen^7^, Laure B. Bindels^1^ and Nathalie M. Delzenne^1*^.

**Affiliation**

^1^Metabolism and Nutrition Research Group, Louvain Drug Research Institute, UCLouvain, Université catholique de Louvain, Brussels, Belgium.

^2^Laboratory of Diabetology, Nutrition and Metabolic disease, Liège, Université de Liège, Belgium.

^3^Pole of Cardiovascular Research, Institut de Recherche Expérimentale et Clinique, Université catholique de Louvain, Brussels, Belgium.

^4^WELBIO- Walloon Excellence in Life Sciences and BIOtechnology, UCLouvain, Université catholique de Louvain, Brussels, Belgium.

^5^Laboratory of Hepatogastroenterology, Institut de Recherche Expérimentale et Clinique, UCLouvain, Université catholique de Louvain, Brussels, Belgium.

^6^ULB Center for Diabetes Research, Université Libre de Bruxelles, and Division of Endocrinology, Erasmus Hospital, Brussels, Belgium.

^7^Pole of Endocrinology, Diabetes and Nutrition, Institut de Recherche Expérimentale et Clinique, UCLouvain, Université catholique de Louvain, Brussels, Belgium.

**1. ADDITIONAL INFORMATION FOR METHODS**

**1.1. Anthropometric characteristics, clinical outcomes and blood parameters**

Weight, height, waist and hip circumference, blood pressure and body composition were measured at baseline and after three months of intervention. Body composition was assessed by bio-impedance devices (BIA 101, Akern, Italy; Biocorpus, Medi Cal, Germany; Tanita BC-418 MA, Tanita, UK). The measure of resistance obtained was used to calculate total body fat according to the method described by Sun et al [2]. Visceral fat area was obtained by abdominal CT-scan. Liver stiffness was performed using FibroScan^®^ (Echosens, Paris, France).

Glycemia, HbA1c, AST, ALT, gGT, total cholesterol, high-density (HDL) and low-density lipoproteins (LDL) cholesterol and triglycerides were measured in fasting plasma samples. The remainder of the blood was centrifuged at 2000 x g for 10 min at 4°C and the plasma was frozen at -80°C. Insulin and C-peptide levels were measured by ELISA kit (Mercodia, Uppsala, Sweden). Homeostasis model assessment of insulin resistance (HOMA-IR) was calculated with the following formula: fasting plasma insulin (in mU/mL) * fasting plasma glucose (in mg/dL) / 405 and Matsuda as described [3]. Dipeptidyl-peptidase IV (DPP-IV) activity was assessed as previously described [4].

**1.2. Gastrointestinal symptoms**

Participants were asked to fill out 100-mm visual analog scales (VAS) describing their gastrointestinal symptoms (nausea, flatulence, cramp, bloating, rumbling and reflux) every week. Each subject completed separate scales, one for each symptom. The scales were scored by measuring the distance (in millimeters) from 0. Results were analyzed using a mixed-effects analysis, followed by a Tukey’s multiple comparisons test on GraphPad Prism v8.1.2.

**1.3. Metabolic measurements**

Plasma insulin concentrations were determined using an ultrasensitive ELISA kit (Mercodia, Uppsala, Sweden). Plasma triglycerides, cholesterol and free fatty acid concentrations were measured using kits coupling enzymatic reaction and spectrophotometric detection of reaction endproducts (Diasys Diagnostic and Systems, Holzheim, Germany). Lipid content was measured in the liver and muscle tissues after extraction with chloroform–methanol according to the Folch method. Briefly, 100 mg of tissue were homogenised in 2 ml of chloroform: methanol (2:1). The chloroform phase was evaporated under nitrogen flux and the dried residue was weighted and solubilised in 1.5 ml of isopropanol. Triglyceride and cholesterol concentrations were measured using a kit coupling an enzymatic reaction and spectrophotometric detection of the final product (Diasys Diagnostic and System, Holzheim, Germany).

**1.4. Molecular biology**

Procedures related to RNA extraction and real-time quantitative, as well as protein extraction and immunoblotting have been previously described [5]. For immunoblotting, membranes were incubated overnight with GLUT4 primary antibody (Abcam, Cambridge, UK) and then incubated with a secondary anti-rabbit antibody (Merck Milliopore, MA, USA). α-tubulin was used as loading control for the liver and muscle proteins, respectively.

**1.5. Quantitative PCR for bacteria**

DNA was extracted from the mouse cecal content, or feces, using a QIAamp DNA Stool Mini Kit (Qiagen, Hildren, Germany) including a bead-beating step. Quantification of total bacteria, Bifidobacterium spp., Roseburia spp., Akkermansia muciniphila and Lactobacillus spp. by qPCR were performed according to the previous procedure using the same primers [6]. Results were analyzed using a two-way ANOVA, followed by a Tukey’s multiple comparisons test on GraphPad Prism v8.1.2.

[1] Hiel, S.*, et al.*, Link between gut microbiota and health outcomes in inulin -treated obese patients: Lessons from the Food4Gut multicenter randomized placebo-controlled trial. *Clin Nutr* 2020.

[2] Sun, S. S.*, et al.*, Development of bioelectrical impedance analysis prediction equations for body composition with the use of a multicomponent model for use in epidemiologic surveys. *Am J Clin Nutr* 2003, *77*, 331-340.

[3] Matsuda, M., DeFronzo, R. A., Insulin sensitivity indices obtained from oral glucose tolerance testing: comparison with the euglycemic insulin clamp. *Diabetes Care* 1999, *22*, 1462-1470.

[4] Olivares, M.*, et al.*, The DPP-4 inhibitor vildagliptin impacts the gut microbiota and prevents disruption of intestinal homeostasis induced by a Western diet in mice. *Diabetologia* 2018, *61*, 1838-1848.

[5] Rodriguez, J.*, et al.*, Discovery of the gut microbial signature driving the efficacy of prebiotic intervention in obese patients. *Gut* 2020.

[6] Neyrinck, A. M.*, et al.*, Chitin-glucan and pomegranate polyphenols improve endothelial dysfunction. *Sci Rep* 2019, *9*, 14150.
